# The Impact of Coronary Calcium Scores on the Development of Myocardial Ischemia: An Analysis of Associated Risk Factors

**DOI:** 10.7759/cureus.94076

**Published:** 2025-10-07

**Authors:** Noorul Hadi, Laila Khan, Ashraf, Asfandiyar, Farooq Ahmad, Mustafa Javaid, Rumman

**Affiliations:** 1 Cardiology, Medical Teaching Institution, Mardan Medical Complex, Mardan, PAK; 2 Radiology, Mardan Medical Complex, Mardan, PAK; 3 Research and Development, Pro-Gene Diagnostics and Research Laboratory, Mardan, PAK; 4 Cardiology, Medical Teaching Institution, Khyber Medical College/Khyber Teaching Hospital, Peshawar, PAK; 5 Internal Medicine, Oxford University Hospitals NHS Foundation Trust, Oxford, GBR; 6 Pharmacovigilance, Association for Community Development, Mardan Medical Complex Teaching Hospital, Mardan, PAK

**Keywords:** atherosclerosis, cardiovascular risk, coronary artery calcium (cac) score, myocardial ischemia, risk stratification

## Abstract

Background: Cardiovascular diseases (CVDs) remain a leading cause of morbidity and mortality globally, with myocardial ischemia (MI) being one of the most critical manifestations of coronary artery disease (CAD). Myocardial ischemia occurs when there is an insufficient supply of oxygen-rich blood to the heart muscle, typically caused by blockages or narrowing in the coronary arteries due to atherosclerosis. The coronary artery calcium (CAC) score, which quantifies calcium deposits in the coronary arteries, has emerged as a valuable tool in predicting cardiovascular events, including myocardial ischemia. Understanding the relationship between CAC scores and myocardial ischemia, along with other risk factors, could improve patient management and help predict adverse cardiovascular outcomes.

Objective: This study aims to evaluate the relationship between CAC scores and the development of myocardial ischemia, as detected through diagnostic imaging methods such as CT angiography and stress echocardiography, and to analyze associated risk factors, including traditional and emerging clinical factors.

Methodology: A prospective, cross-sectional observational study was conducted in the Departments of Cardiology and Radiology at Mardan Medical Complex between February 2023 and June 2025. Diagnostic methods such as CT angiography and stress echocardiography were used to assess the presence of myocardial ischemia. Demographic data, clinical characteristics, comorbidities, and CAC scores were collected. Statistical analyses, including two-way analysis of variance (ANOVA), Mann-Whitney U tests, and decision tree modeling, were used to explore the relationship between CAC scores and myocardial ischemia, along with various risk factors such as age, gender, BMI, smoking history, and comorbidities.

Results: The study included 129 patients, with 74 (57.36%) diagnosed with myocardial ischemia. A strong correlation was observed between elevated CAC scores and ischemia, with ischemic patients displaying significantly higher CAC scores (671.92 ± 185.82) compared to nonischemic patients (246.36 ± 98.51). The overall CAC scores for individual coronary arteries were 73.57 ± 39.24 for the left main artery (LMA), 196.19 ± 104.63 for the left anterior descending artery (LAD), 122.62 ± 65.40 for the left circumflex artery (LCX), and 98.10 ± 52.32 for the right coronary artery (RCA), while the total CAC score averaged 490.48 ± 261.58. Decision tree analysis identified critical cutoff values for predicting ischemia, including 403.0 for total CAC, 161.2 for LAD, 100.75 for LCX, 80.6 for RCA, and 60.45 for LMA. Additionally, risk factors such as hypertension, diabetes, and smoking were associated with higher CAC scores, particularly among ischemic patients.

Conclusion: CAC scoring is an essential tool for evaluating the risk of myocardial ischemia and cardiovascular events, complementing traditional risk factors to improve stratification and enable more focused interventions for patients.

## Introduction

Cardiovascular diseases (CVDs), particularly coronary artery disease (CAD), significantly contribute to global morbidity and mortality rates [1]. Among the many clinical manifestations of CAD, myocardial ischemia (MI) is critical, as it can lead to severe cardiovascular events, including heart attacks and heart failure [2,3], if not addressed in a timely manner. Myocardial ischemia primarily occurs due to inadequate blood flow to the heart muscle, often as a result of obstruction or narrowing of the coronary arteries caused by atherosclerotic plaques [4]. Identifying individuals at risk for myocardial ischemia is crucial for preventive management and the timely initiation of appropriate interventions.

The assessment of CAD has evolved considerably over recent decades, driven largely by advancements in imaging technology [5]. Noninvasive imaging modalities are now indispensable tools in the early detection and diagnosis of CAD [6]. The coronary artery calcium (CAC) score, derived from high-resolution computed tomography (CT) scans, has gained prominence for its utility in predicting cardiovascular risk [7]. The CAC score quantifies calcium deposits within the coronary arteries, serving as a reliable biomarker of atherosclerotic burden [8]. It enables assessment of both the presence and severity of CAD, which has significant prognostic implications for myocardial ischemia [9], characterized by diminished blood supply to the myocardium [10].

Several studies have demonstrated a strong correlation between elevated CAC scores and adverse cardiovascular outcomes [11]. In a meta-analysis involving more than 34,000 subjects, increased CAC was associated with a higher risk for nonfatal myocardial infarction (MI) and mortality [12]. Patients with CAC scores above established thresholds exhibited a markedly higher incidence of cardiovascular events, reinforcing the utility of this scoring in risk stratification related to coronary artery blockage or occlusion.

Despite its advantages, the CAC score has limitations. Notably, it cannot distinguish between stable and unstable plaques [13], which may oversimplify a complex disease process. However, its strong predictive ability for ischemic events has led many experts to recommend its use [14], especially in cases where traditional risk factors do not fully represent a patient’s risk profile. Metrics such as blood pressure and cholesterol can sometimes lead to misclassification of risk, making CAC scoring an important adjunctive tool.

In addition to CAC scoring, other established risk factors, such as hypertension, diabetes mellitus, dyslipidemia, and lifestyle factors including smoking and poor dietary habits [15,16], play significant roles in the development of myocardial ischemia. These conventional risks interact with pathological mechanisms such as endothelial dysfunction, chronic inflammation, and the formation and progression of atherosclerotic plaques. Emerging research also highlights nontraditional risk factors, such as genetic predisposition and chronic psychological stress, which may influence the pathophysiology of myocardial ischemia [17,18] alongside CAC load.

Accordingly, the multifactorial nature of CAD necessitates a comprehensive approach to patient assessment. Imaging modalities such as CT angiography and stress echocardiography are routinely employed for diagnosing myocardial ischemia, ensuring thorough evaluation of both functional and anatomical abnormalities within the coronary circulation [19,20]. Integrating CAC scoring into current diagnostic practices enhances the stratification of high-risk individuals, which can guide treatment decisions toward more tailored and effective interventions.

Studies indicate that individuals with high CAC scores have a greater likelihood of silent myocardial ischemia, which often goes undetected in clinical settings [21]. Conversely, individuals with low or zero CAC scores can often be reassured of their low likelihood of significant CAD, potentially avoiding unnecessary invasive testing.

Moreover, the stability of coronary plaques, as assessed through CAC scores, can inform appropriate management strategies ranging from lifestyle changes and pharmacotherapy to more aggressive interventions [22,23]. Personalized treatment plans developed through a detailed understanding of an individual’s risk factors, facilitated by CAC evaluations, can improve management outcomes and prevent progression to symptomatic ischemic heart disease.

Recent studies continue to clarify the relationship between CAC burden and ischemic outcomes, prompting efforts to refine the clinical application of CAC scoring. Current research seeks to define precise thresholds that link CAC scores with ischemic risk and its associated factors, thereby improving the prediction of adverse events across diverse populations. Variability in patient response underscores the importance of individualized management strategies. With this knowledge, clinicians can employ CAC scoring to optimize care for high-risk individuals, ultimately improving cardiovascular outcomes in an era of rising disease prevalence.

## Materials and methods

Study design and setting

A prospective, cross-sectional observational study was conducted in the Departments of Cardiology and Radiology at Mardan Medical Complex between February 2023 and June 2025. The study aimed to evaluate the relationship between coronary artery calcium CAC scores and the development of myocardial ischemia, as detected by diagnostic imaging techniques such as CT angiography and stress echocardiography. Additionally, the study sought to analyze associated cardiovascular risk factors within this cohort. A convenience sampling approach was used to recruit participants presenting to the hospital with suspected CAD or undergoing routine cardiovascular screening during the study period.

Inclusion and exclusion criteria

**Figure 1:**
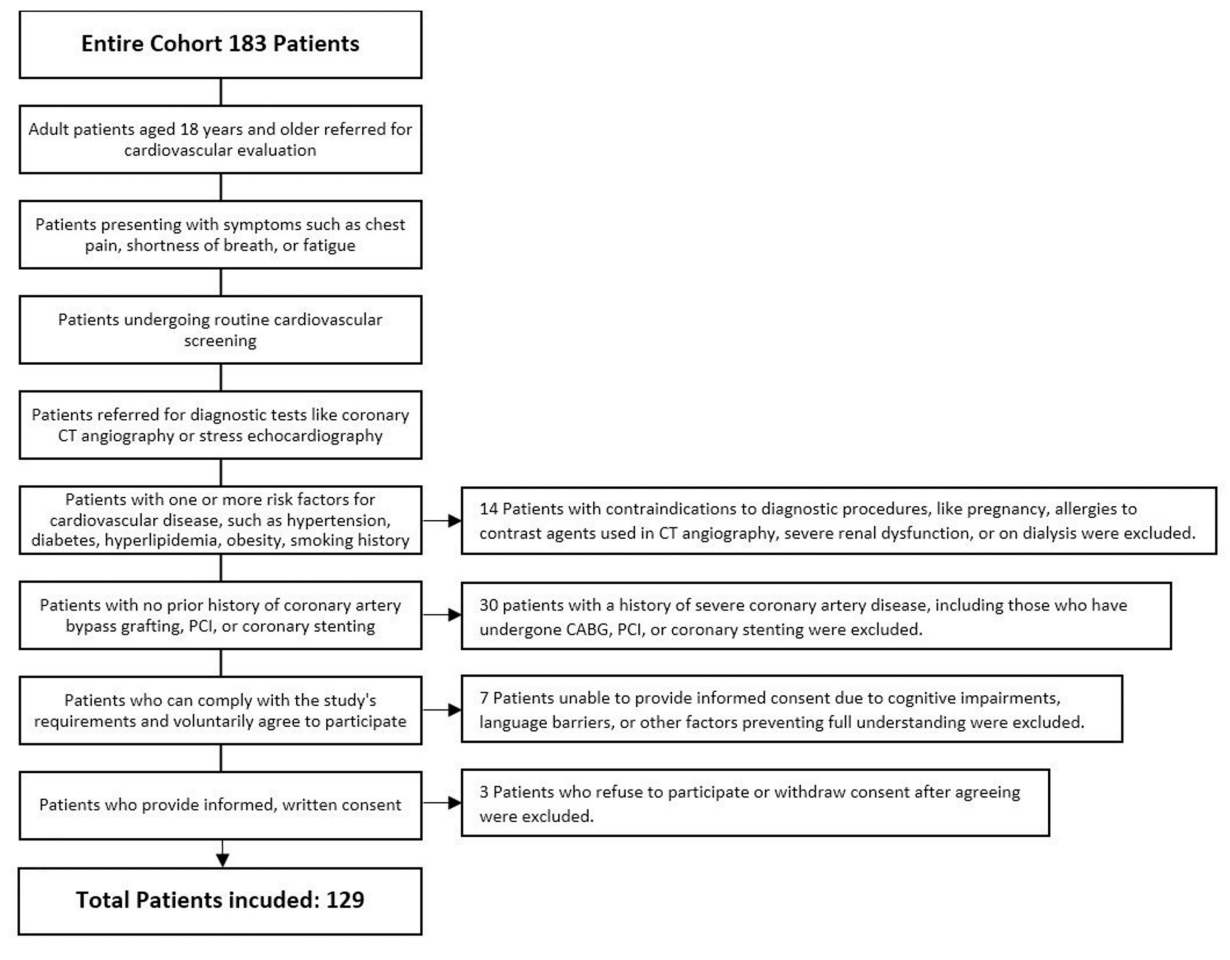
Inclusion and exclusion criteria of participants in the present study This flowchart illustrates the inclusion and exclusion criteria used to select the study participants. The initial cohort consisted of 183 patients who were referred for cardiovascular evaluation.

The following flow chart represents the inclusion and exclusion criteria (Figure 1).

Sample size calculation

A power analysis was conducted using G*Power version 3.1 (Heinrich Heine University Düsseldorf, Düsseldorf, Germany) [22] to determine the adequacy of the sample size for detecting meaningful associations between CAC scores and myocardial ischemia. The total sample size of 129 participants, including 74 (57.36%) with myocardial ischemia and 55 (42.64%) without, was found to provide sufficient statistical power. Assuming a moderate effect size (Cohen’s d = 0.5), an alpha level of 0.05, and a statistical power of 0.80, the sample size was adequate to evaluate the relationship between CAC scores and the presence of ischemia, ensuring reliable results while accounting for potential dropouts or missing data.

Demographic and clinical data collection

Demographic and clinical data were collected from participants’ medical records and structured interviews, assessing various variables. Demographic information, including age, gender, and body mass index (BMI), was recorded. Cardiovascular risk factors were evaluated, such as a medical history of hypertension, hyperlipidemia, diabetes, smoking, chronic kidney disease, atrial fibrillation, and chronic heart failure. Cardiac conditions were also assessed, including a history of ischemia, MI, collateral circulation, and cardiomyopathy, with further classification into restrictive, dilated, and hypertrophic types. Additionally, comorbidities such as valvular heart disease were noted. Participants’ cardiac function was evaluated by left ventricular ejection fraction (LVEF), with measurements categorized into 50-60% and 40-50% ranges. Evidence of right ventricular dysfunction was recorded, along with data on exercise and pharmacological stress durations. CT angiography grades were also assessed, indicating varying levels of CAD severity. Lastly, lipid profiles, including total cholesterol, triglycerides, high-density lipoprotein (HDL), and low-density lipoprotein (LDL) cholesterol, were analyzed using a Cobas® c311 automated biochemistry analyzer (Roche Diagnostics, Germany), and CAC scores were recorded to assess the cardiovascular risk of participants.

Diagnostic imaging and CAC scoring

All participants underwent CT angiography to measure the total CAC score and assess the distribution of calcification in the major coronary arteries. The CAC scores were measured individually for each artery, including the left main artery (LMA), the left anterior descending artery (LAD), the left circumflex artery (LCX), and the right coronary artery (RCA). The calcium score for the LMA was evaluated to assess calcification in this critical vessel. The CAC score for the LAD was obtained to determine the burden of calcium in this major artery, which supplies a significant portion of the heart. The LCX calcium score was assessed for its contribution to the overall coronary calcification burden, while the RCA CAC score was measured to determine the extent of calcium buildup in the right coronary artery. The total CAC score was calculated by summing the individual scores from all coronary arteries. The images were analyzed using the Agatston scoring method [23], which quantifies the total calcium burden based on the number, density, and size of calcified plaques in the arteries.

Agatston Scoring Formula

The Agatston score for each coronary lesion is calculated using the following formula:

$\text{Agatston score}=(\text{Area of calcification}\times \text{Density factor})$

where the area of calcification is the area (in square millimeters) of each calcified lesion detected on the CT scan, and the density factor is the weighted score based on the Hounsfield unit (HU) of each lesion. The density factor is categorized as given in Table 1.

**Table 1:** Density factors

Hounsfield Unit (HU) Range	Density Factor
1–100 HU	1
101–300 HU	2
301–400 HU	3
>400 HU	4

The total CAC score, along with the individual artery scores (LMA, LAD, LCX, and RCA), was recorded and used in subsequent analyses to explore its relationship with myocardial ischemia (Table 2).

**Table 2:** An example of how total CAC score was calculated LMA: left main artery, LAD: left anterior descending artery, LCX: left circumflex artery, RCA: right coronary artery, HU: Hounsfield unit, CAC: coronary artery calcium.

Calculation
LMA
Lesion 1: Area = 50 mm², HU = 120 → Score = 100
Lesion 2: Area = 30 mm², HU = 350 → Score = 90
Lesion 3: Area = 40 mm², HU = 450 → Score = 160
Total LMA score = 100 + 90 + 160 = 350
LAD
Lesion 1: Area = 60 mm², HU = 130 → Score = 120
Lesion 2: Area = 25 mm², HU = 300 → Score = 75
Lesion 3: Area = 50 mm², HU = 420 → Score = 200
Total LAD score = 120 + 75 + 200 = 395
LCX
Lesion 1: Area = 40 mm², HU = 110 → Score = 110
Lesion 2: Area = 35 mm², HU = 370 → Score = 105
Lesion 3: Area = 45 mm², HU = 410 → Score = 180
Total LCX score = 110 + 105 + 180 = 395
RCA
Lesion 1: Area = 30 mm², HU = 140 → Score = 140
Lesion 2: Area = 45 mm², HU = 310 → Score = 135
Lesion 3: Area = 60 mm², HU = 400 → Score = 240
Total RCA score = 140 + 135 + 240 = 515
Total CAC score = 350 (LMA) + 395 (LAD) + 395 (LCX) + 515 (RCA) = 1655

Assessment of myocardial ischemia

Myocardial ischemia was diagnosed using two diagnostic imaging methods. First, participants underwent stress echocardiography, which involved monitoring changes in heart function under physical or pharmacologic stress, such as treadmill exercise or adenosine infusion. Ischemia was identified by abnormal wall motion. Additionally, for confirmation, or if stress echocardiography was not suitable, coronary CT angiography was performed to assess the presence of coronary artery stenosis. This imaging method helped identify significant blockages or narrowing in the coronary arteries that could contribute to ischemia.

Statistical analysis

Statistical analyses were conducted using R (version 4.5.1, R Foundation for Statistical Computing, Vienna, Austria). Descriptive statistics were used to summarize the demographic, clinical, and laboratory data, with continuous variables presented as means and standard deviations (SD) and categorical variables expressed as frequencies and percentages. To examine the relationship between the total CAC score and the LMA, LAD, LCX, and RCA with ischemia status and other variables, boxplots were created for visual representation. A two-way analysis of variance (ANOVA) was used to explore the associations between CAC scores, ischemia, and various factors, while the Mann-Whitney U test was specifically applied to assess differences in CAC scores with respect to ischemia. Scatter plots were generated to examine the relationships between CAC scores, ischemia, and lipid profile parameters, with R-squared values calculated to measure the strength of these relationships. Decision trees were utilized to determine the cutoff values for the total CAC score, LMA, LAD, LCX, and RCA in predicting the presence of ischemia, with the Gini index used to rank the importance of predictors within the decision tree model. A p-value of less than 0.05 was considered statistically significant.

Ethical considerations

This study was conducted in accordance with ethical guidelines approved by the Institutional Review Board (IRB) of Mardan Medical Complex. Informed consent was obtained from all participants prior to inclusion in the study. The consent form explained the purpose of the study, the procedures involved, and potential risks. Participants were assured that their confidentiality would be maintained and that their data would be anonymized and stored securely. The study did not incur any additional costs for participants, as all diagnostic imaging and laboratory tests were covered by the research department.

## Results

The study included 129 patients, with an average age of 59.64 ± 9.43 years, predominantly male (73 patients, 56.59%) and overweight, with a mean BMI of 28.86 ± 3.92. A majority of patients (85, 65.89%) were non-smokers. Comorbidities were common, with hypertension affecting 81 patients (62.79%), hyperlipidemia in 60 (46.51%), and diabetes in 56 (43.41%). Chronic kidney disease was present in 32 patients (24.81%), atrial fibrillation in 12 (9.30%), and chronic heart failure in 6 (4.65%). Ischemia was present in 74 patients (57.36%), while 49 (37.98%) had a history of MI. Collateral circulation was observed in 63 patients (48.84%), and cardiomyopathy was found in 49 patients (37.98%), with restrictive, dilated, and hypertrophic types affecting 19 (14.73%), 16 (12.40%), and 14 (10.85%), respectively. Valvular heart disease affected 63 patients (48.84%) (Table 3).

**Table 3:** Demographic, clinical, and cardiac characteristics of the study population

Characteristics	Values
All patients, n (%)	129(100%)
Age, mean ± standard deviation	59.64 ± 9.43
Gender, n (%)	
Male	73(56.59%)
Female	56(43.41%)
Body mass index (BMI), mean ± standard deviation	28.86 ± 3.92
Smoking history, n (%)	
No	85(65.89%)
Yes	44(34.11%)
Comorbidities, n (%)	
None	32(24.81%)
Hypertension	81(62.79%)
Hyperlipidemia	60(46.51%)
Diabetes	56(43.41%)
Chronic kidney disease	32(24.81%)
Atrial fibrillation	12(9.30%)
Chronic heart failure	6(4.65%)
Cardiac conditions	
Ischemia, n (%)	
Yes	74(57.36%)
No	55(42.64%)
History of myocardial infarction, n (%)	
No	80 (62.02%)
Yes	49(37.98%)
Collateral circulation, n (%)	
No	66(51.16%)
Yes	63(48.84%)
Cardiomyopathy, n (%)	
No	80(62.02%)
Restrictive	19(14.73%)
Dilated	16(12.40%)
Hypertrophic	14(10.85%)
Valvular heart disease, n (%)	
No	66(51.16%)
Yes	63(48.84%)

This table presents the characteristics related to cardiac function and stress test results in the study population of 129 patients. Left ventricular ejection fraction (LVEF) was measured, with 62.02% of patients (80 patients) falling within the 50-60% range and 37.98% (49 patients) having an LVEF between 40-50%, indicating varying degrees of left ventricular function. Right ventricular dysfunction was present in 23.26% of the patients (30 patients), while the majority, 76.74% (99 patients), did not have right ventricular dysfunction. In terms of exercise or pharmacological stress duration, 57.36% (74 patients) had stress durations of 7-9 minutes, while 42.64% (55 patients) were able to exercise or undergo pharmacological stress for 10-12 minutes. The CT angiography results showed that 2.32% (3 patients) had Grade 2 CAD, 40.31% (52 patients) had Grade 3, and the majority, 57.36% (74 patients), were classified as Grade 4, indicating severe CAD (Table 4).

**Table 4:** Cardiac function and stress test characteristics of the study population

Characteristics	Values, n (%)
All patients	129(100%)
Left ventricular ejection fraction (LVEF) measurement	
50-60%	80(62.02%)
40-50%	49(37.98%)
Evidence of right ventricular dysfunction	
No	99(76.74%)
Yes	30(23.26%)
Exercise duration	
7-9 minutes	74(57.36%)
10-12 minutes	55(42.64%)
CT angiograph grades	
Grade 2	3(2.32%)
Grade 3	52(40.31%)
Grade 4	74(57.36%)

The total CAC score had a mean of 490.48 ± 261.58, indicating a moderate level of coronary artery calcification. Specific coronary artery territories showed varying levels of calcification, with the LAD showing the highest mean CAC score of 196.19 ± 104.63, followed by the LCX at 122.62 ± 65.40 and the RCA at 98.10 ± 52.32. The LMA had a mean CAC score of 73.57 ± 39.24. Regarding lipid profiles, total cholesterol averaged 262.79 ± 45.83, which is elevated and suggests a high cardiovascular risk. Triglyceride levels were also high at 224.45 ± 80.82, while HDL cholesterol levels, a protective factor, were relatively low at 43.84 ± 6.33. LDL cholesterol, a major contributor to atherosclerosis, had an average of 131.68 ± 50.55 (Table 5).

**Table 5:** Lipid profile and coronary artery calcium scores of the study population

Characteristics	Values (mean ± standard deviation)
Coronary artery calcium scores across different coronary arteries	
Total coronary artery calcium score	490.48±261.58
Left main artery (LMA)	73.57±39.24
Left anterior descending artery (LAD)	196.19±104.63
Left circumflex artery (LCX)	122.62±65.40
Right coronary artery (RCA)	98.10±52.32
Lipid profile	
Total cholesterol	262.79 ± 45.83
Triglyceride levels	224.45 ± 80.82
HDL cholesterol levels	43.84 ± 6.33
LDL cholesterol levels	131.68 ± 50.55

**Figure 2:**
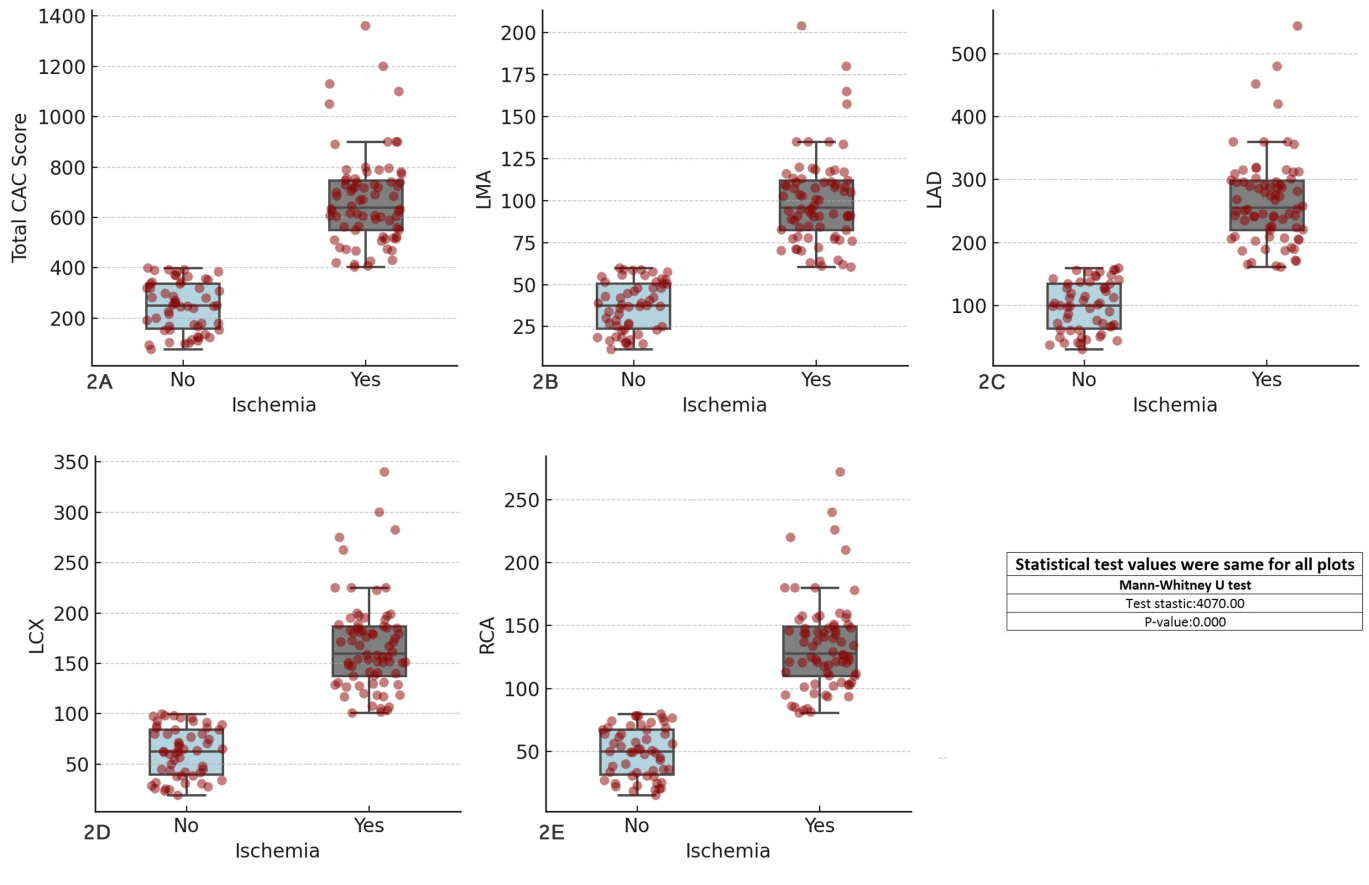
Comparison of coronary artery calcium (CAC) scores across major coronary arteries in patients with and without ischemia Each boxplot compares the distribution of CAC scores for the specified coronary artery (total CAC, LMA, LAD, LCX, RCA) between patients with and without ischemia (Yes/No). The boxplots display the interquartile range (IQR), with the median indicated, and individual data points overlaid as red dots. The Mann–Whitney U test was used for comparisons. (A) Total CAC score vs ischemia, (B) left main artery (LMA) CAC score vs ischemia, (C) left anterior descending artery (LAD) CAC score vs ischemia, (D) left circumflex artery (LCX) CAC score vs ischemia, (E) right coronary artery (RCA) CAC score vs ischemia.

A strong association was found between elevated CAC scores and ischemia, with ischemic patients exhibiting significantly higher CAC scores (671.92 ± 185.82) compared with non-ischemic patients (246.36 ± 98.51). The CAC scores for individual coronary arteries were as follows: 73.57 ± 39.24 for the LMA, 196.19 ± 104.63 for the LAD, 122.62 ± 65.40 for the LCX, and 98.10 ± 52.32 for the RCA. The average total CAC score was 490.48 ± 261.58, with all comparisons showing statistically significant differences (p < 0.001). Higher CAC scores were consistently observed in ischemic patients (Figure 2).

**Figure 3:**
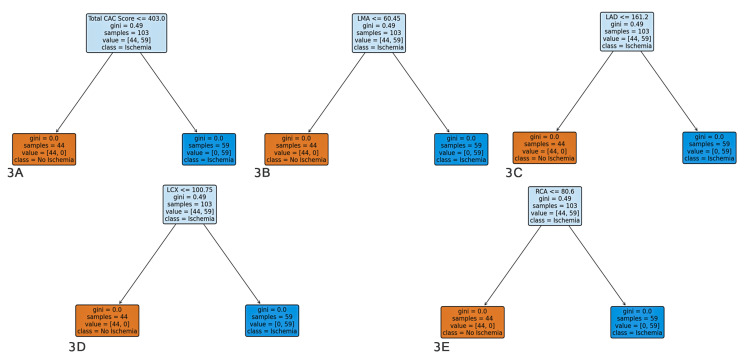
Decision tree analysis of coronary artery calcium (CAC) scores in predicting myocardial ischemia status Each plot shows the decision tree analysis for predicting myocardial ischemia (Yes/No) based on CAC scores for the indicated coronary artery segment. The decision tree splits data based on thresholds of CAC scores to predict the likelihood of ischemia. The decision nodes represent CAC score thresholds, and the branches indicate the corresponding predicted ischemia status. (A) Total CAC score vs ischemia status, (B) left main artery (LMA) CAC score vs ischemia status, (C) left anterior descending artery (LAD) CAC score vs ischemia status, (D) left circumflex artery (LCX) CAC score vs ischemia status, (E) right coronary artery (RCA) CAC score vs ischemia status.

The decision tree models determined the following cutoff values for CAC scores to predict ischemia: 403.0 for the total CAC score, 60.45 for the LMA, 161.7 for the LAD, 100.75 for the LCX, and 56.8 for the right coronary artery (RCA) (Figure 3).

**Figure 4:**
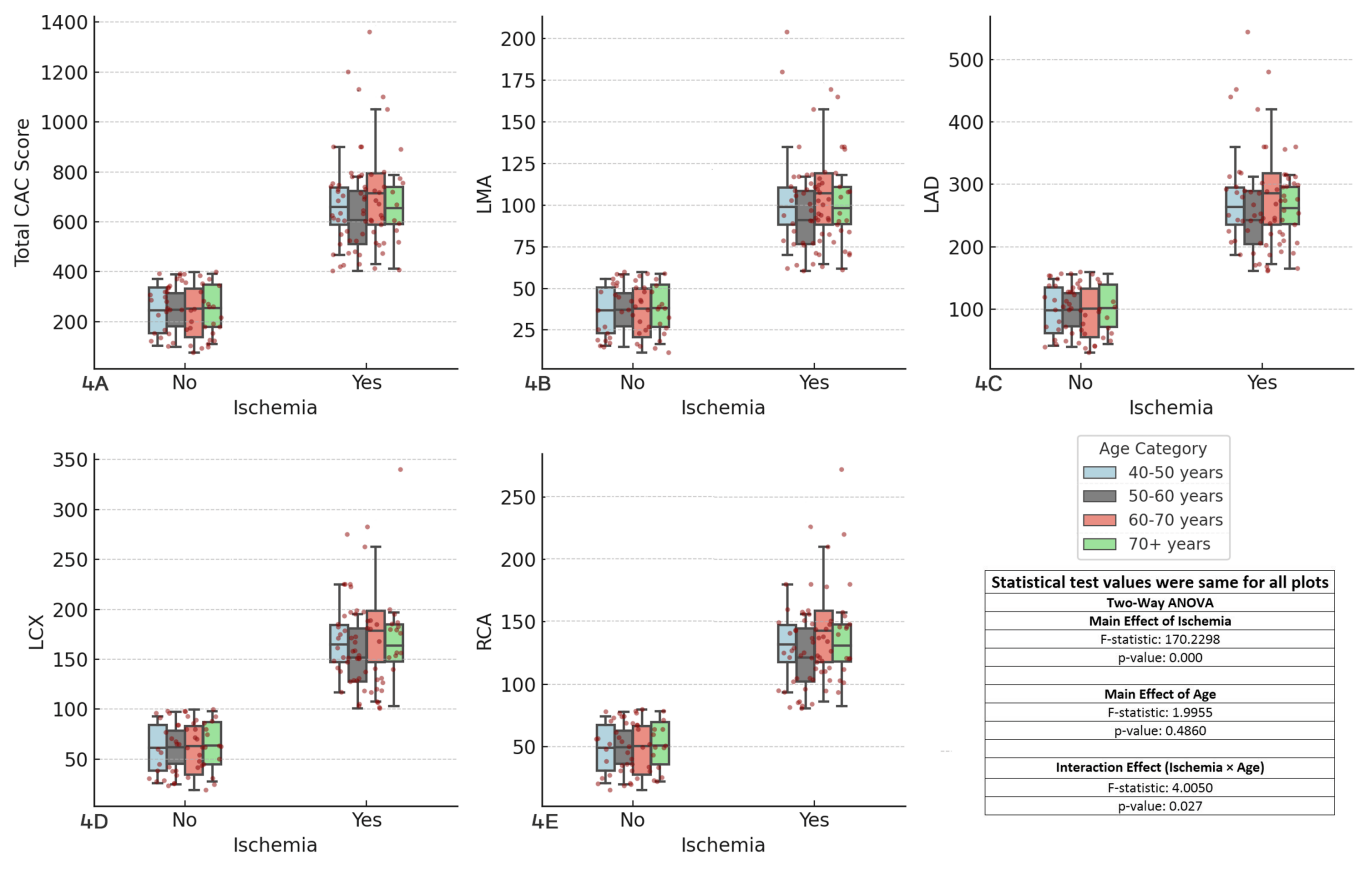
Comparison of coronary artery calcium (CAC) scores across major coronary arteries and ischemia status by age groups Each boxplot compares the distribution of CAC scores for the specified coronary artery (total CAC, LMA, LAD, LCX, RCA) between patients with and without ischemia (Yes/No), considering age as a factor. The boxplots display the interquartile range (IQR), with the median indicated, and individual data points overlaid as red dots. The two-way ANOVA test was used for comparisons. (A) Total CAC score vs ischemia and age, (B) left main artery (LMA) CAC score vs ischemia and age, (C) left anterior descending artery (LAD) CAC score vs ischemia and age, (D) left circumflex artery (LCX) CAC score vs ischemia and age, (E) right coronary artery (RCA) CAC score vs ischemia and age.

The results showed significant main effects of ischemia (F-statistic = 170.2298, p < 0.001) across all coronary arteries, while the main effect of age was not significant (F-statistic = 1.9955, p = 0.4860). However, the interaction effect between ischemia and age was significant (F-statistic = 4.0500, p = 0.027), indicating that the combination of ischemia and aging influences CAC scores. The 60-70 and ≥70 years age groups exhibited the highest CAC scores, particularly in ischemic patients, suggesting that advancing age is a critical factor in the development of CAD. These findings highlight the importance of incorporating both age and ischemia status in risk stratification for CAD (Figure 4).

**Figure 5:**
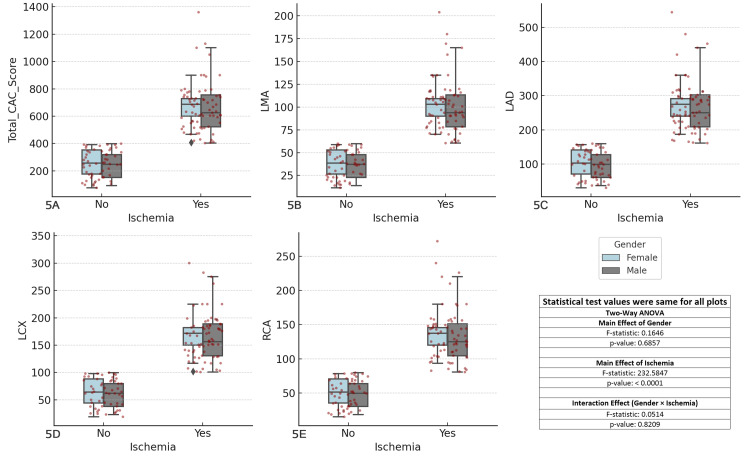
Comparison of coronary artery calcium (CAC) scores across major coronary arteries and ischemia status by gender Each boxplot compares the distribution of CAC scores for the specified coronary artery (total CAC, LMA, LAD, LCX, RCA) between patients with and without ischemia (Yes/No), considering gender as a factor. The boxplots display the interquartile range (IQR), with the median indicated, and individual data points overlaid as red dots. The two-way ANOVA test was used for comparisons. (A) Total CAC score vs ischemia and gender, (B) left main artery (LMA) CAC score vs ischemia and gender, (C) left anterior descending artery (LAD) CAC score vs ischemia and gender, (D) left circumflex artery (LCX) CAC score vs ischemia and gender, (E) right coronary artery (RCA) CAC score vs ischemia and gender.

The results revealed significant main effects of ischemia (F-statistic = 232.5847, p < 0.0001) across all coronary arteries, while the main effect of gender was not significant (F-statistic = 0.1646, p = 0.6857). The interaction effect between gender and ischemia was also not significant (F-statistic = 0.0514, p = 0.8209). These findings suggest that ischemia has a significant impact on CAC scores, but gender does not appear to significantly influence the scores or their relationship with ischemia in the studied coronary arteries. The results highlight the primary importance of ischemia in the development of CAD, with gender showing no notable difference in CAC scores (Figure 5).

**Figure 6:**
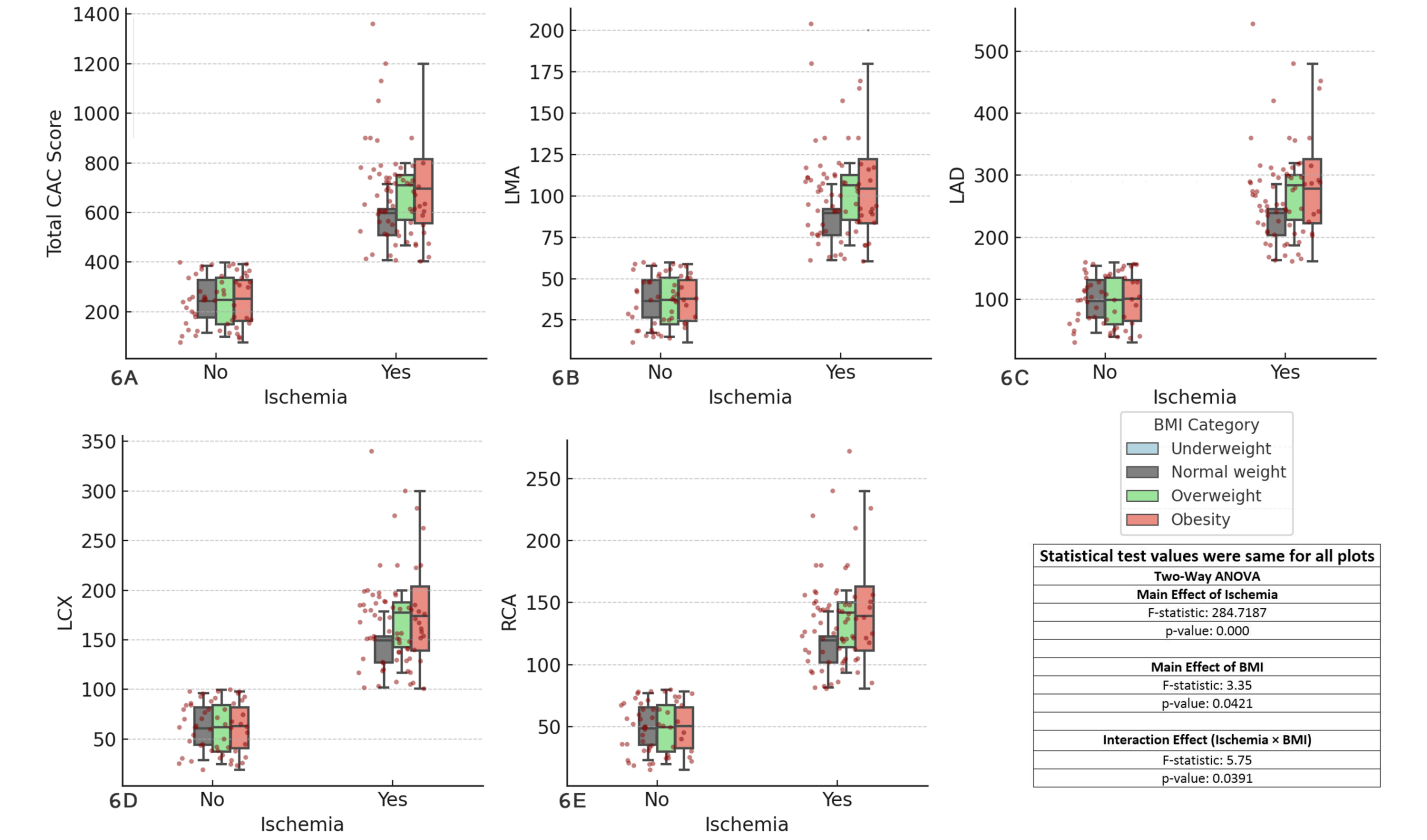
Comparison of coronary artery calcium (CAC) scores across major coronary arteries and ischemia status by BMI categories Each boxplot compares the distribution of CAC scores for the specified coronary artery (total CAC, LMA, LAD, LCX, RCA) between patients with and without ischemia (Yes/No), considering BMI as a factor. The boxplots display the interquartile range (IQR), with the median indicated, and individual data points overlaid as red dots. The two-way ANOVA test was used for comparisons. (A) Total CAC score vs ischemia and BMI, (B) left main artery (LMA) CAC score vs ischemia and BMI, (C) left anterior descending artery (LAD) CAC score vs ischemia and BMI, (D) left circumflex artery (LCX) CAC score vs ischemia and BMI, (E) right coronary artery (RCA) CAC score vs ischemia and BMI.

The results showed significant main effects of ischemia (F-statistic = 284.7187, p < 0.001) across all coronary arteries. The main effect of BMI was marginally significant (F-statistic = 3.35, p = 0.0421), suggesting that BMI has a modest but noteworthy influence on CAC scores. Additionally, the interaction effect between ischemia and BMI was also significant (F-statistic = 5.75, p = 0.0391), indicating that the relationship between ischemia and CAC scores varies across different BMI categories. These findings emphasize the importance of considering BMI, along with ischemia, in assessing CAD risk (Figure 6).

**Figure 7:**
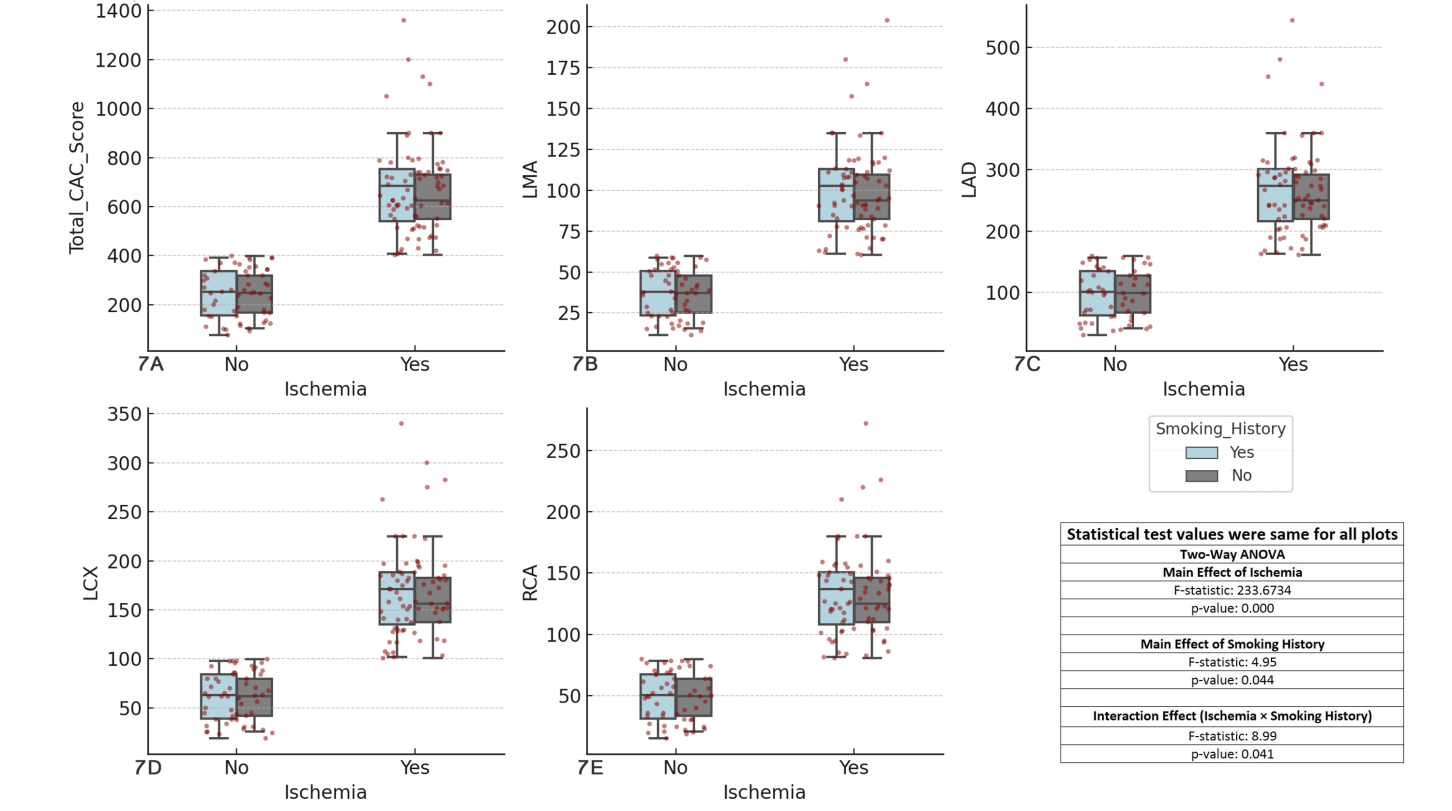
Comparison of coronary artery calcium (CAC) scores across major coronary arteries and ischemia status by smoking history Each boxplot compares the distribution of CAC scores for the specified coronary artery (total CAC, LMA, LAD, LCX, RCA) between patients with and without ischemia (Yes/No), considering smoking as a factor. The boxplots display the interquartile range (IQR), with the median indicated, and individual data points overlaid as red dots. The two-way ANOVA test was used for comparisons. (A) Total CAC score vs ischemia and smoking history, (B) left main artery (LMA) CAC score vs ischemia and smoking history, (C) left anterior descending artery (LAD) CAC score vs ischemia and smoking history, (D) left circumflex artery (LCX) CAC score vs ischemia and smoking history, (E) right coronary artery (RCA) CAC score vs ischemia and smoking history.

The results showed significant main effects of ischemia (F-statistic = 233.6734, p < 0.001) across all coronary arteries. The main effect of smoking history was also significant (F-statistic = 4.95, p = 0.044), suggesting that smoking history influences CAC scores. Additionally, the interaction effect between ischemia and smoking history was significant (F-statistic = 8.99, p = 0.041), indicating that the relationship between ischemia and CAC scores is influenced by whether a person has a smoking history. These findings emphasize the importance of considering smoking history alongside ischemia in the clinical assessment of CAD risk (Figure 7).

**Figure 8:**
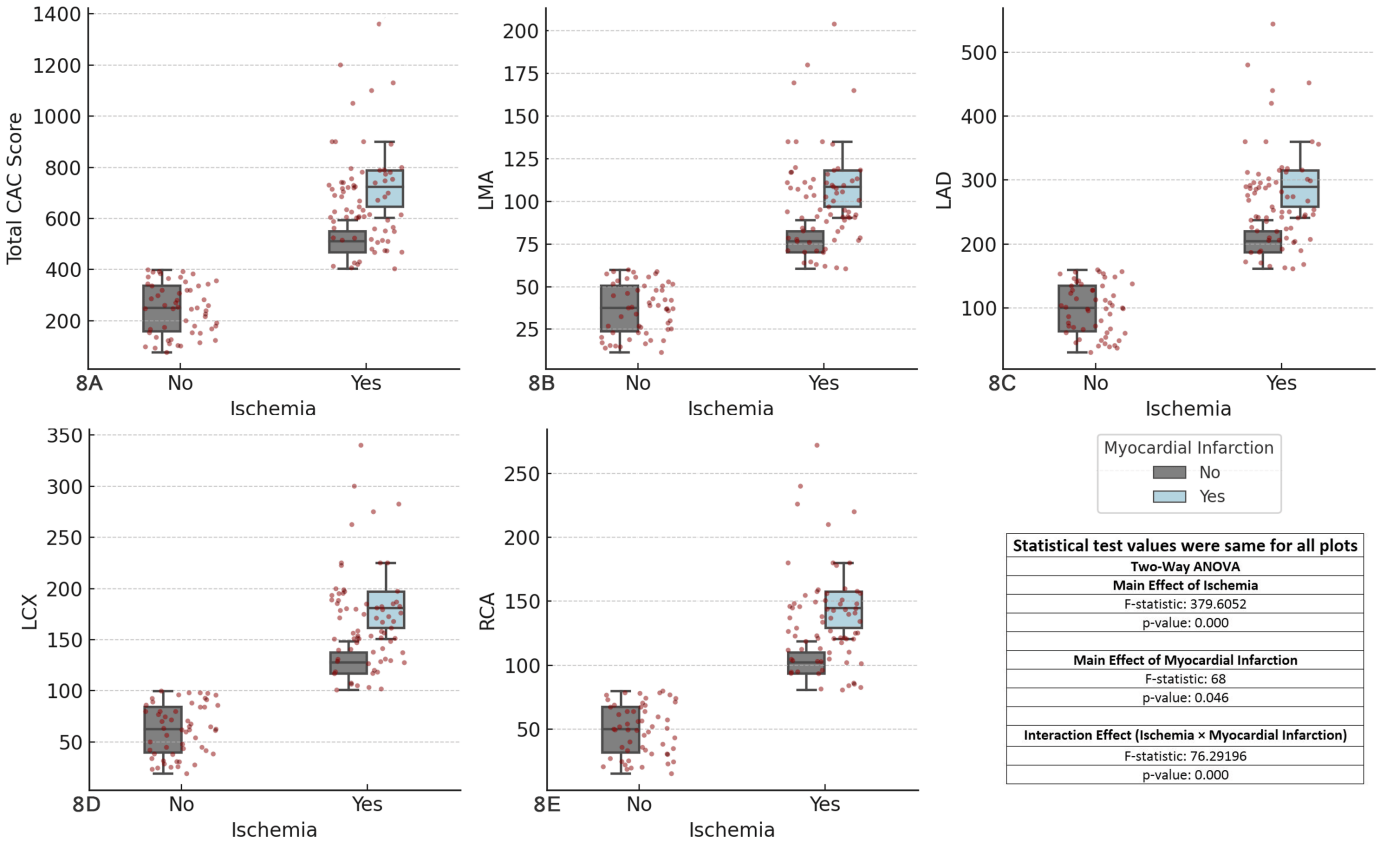
Comparison of coronary artery calcium (CAC) scores across major coronary arteries, ischemia, and myocardial infarction status Each boxplot compares the distribution of CAC scores for the specified coronary artery (total CAC, LMA, LAD, LCX, RCA) between patients with and without ischemia (Yes/No), considering myocardial infarction status as a factor. The boxplots display the interquartile range (IQR), with the median indicated, and individual data points overlaid as red dots. The two-way ANOVA test was used for comparisons. (A) Total CAC score vs ischemia and myocardial infarction, (B) left main artery (LMA) CAC score vs ischemia and myocardial infarction, (C) left anterior descending artery (LAD) CAC score vs ischemia and myocardial infarction, (D) left circumflex artery (LCX) CAC score vs ischemia and myocardial infarction, (E) right coronary artery (RCA) CAC score vs ischemia and myocardial infarction.

The results revealed significant main effects of ischemia (F-statistic = 379.6052, p < 0.001) across all coronary arteries. The main effect of MI was also significant (F-statistic = 68.0000, p-value = 0.046), indicating that MI influences CAC scores. Additionally, the interaction effect between ischemia and MI was highly significant (F-statistic = 76.2919, p < 0.001), suggesting that the relationship between ischemia and CAC scores is influenced by the presence of MI. These findings emphasize the critical role of both ischemia and MI in CAD and the need for comprehensive assessment of both factors in clinical practice (Figure 8).

**Figure 9:**
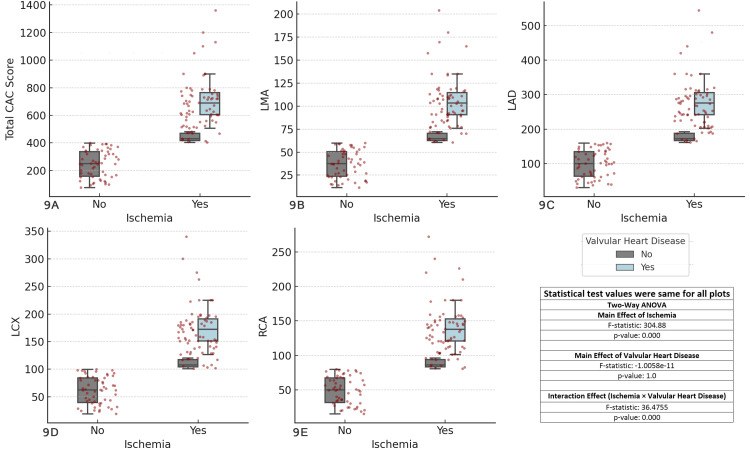
Comparison of coronary artery calcium (CAC) scores across major coronary arteries, ischemia status, and valvular heart disease Each boxplot compares the distribution of CAC scores for the specified coronary artery (total CAC, LMA, LAD, LCX, RCA) between patients with and without ischemia (Yes/No), considering valvular heart disease as a factor. The boxplots display the interquartile range (IQR), with the median indicated, and individual data points overlaid as red dots. The two-way ANOVA test was used for comparisons. (A) Total CAC score vs ischemia and valvular heart disease, (B) left main artery (LMA) CAC score vs ischemia and valvular heart disease, (C) left anterior descending artery (LAD) CAC score vs ischemia and valvular heart disease, (D) left circumflex artery (LCX) CAC score vs ischemia and valvular heart disease, (E) right coronary artery (RCA) CAC score vs ischemia and valvular heart disease.

The results revealed significant main effects of ischemia (F-statistic = 304.8888, p < 0.001) across all coronary arteries, indicating that ischemia strongly influences CAC scores. However, the main effect of valvular heart disease was not significant (F-statistic = 1.0058e-11, p = 1.0), suggesting that it does not independently affect CAC scores. On the other hand, the interaction effect between ischemia and valvular heart disease was significant (F-statistic = 36.4755, p < 0.001), highlighting that the relationship between ischemia and CAC scores is modified by the presence of valvular heart disease. These findings emphasize the critical role of ischemia in CAD and suggest the importance of considering both ischemia and valvular heart disease together in clinical assessments (Figure 9).

**Figure 10:**
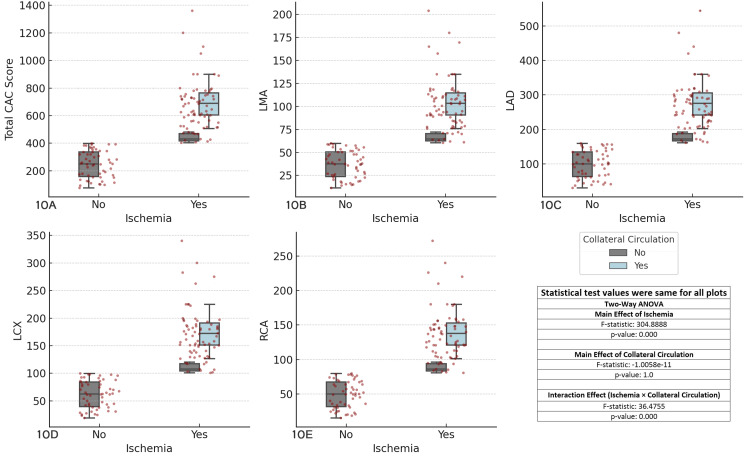
Comparison of coronary artery calcium (CAC) scores across major coronary arteries, ischemia status, and collateral circulation Each boxplot compares the distribution of CAC scores for the specified coronary artery (total CAC, LMA, LAD, LCX, RCA) between patients with and without ischemia (Yes/No), considering collateral circulation as a factor. The boxplots display the interquartile range (IQR), with the median indicated, and individual data points overlaid as red dots. The two-way ANOVA test was used for comparisons. (A) Total CAC score vs ischemia and collateral circulation, (B) left main artery (LMA) CAC score vs ischemia and collateral circulation, (C) left anterior descending artery (LAD) CAC score vs ischemia and collateral circulation, (D) left circumflex artery (LCX) CAC score vs ischemia and collateral circulation, (E) right coronary artery (RCA) CAC score vs ischemia and collateral circulation.

The results revealed significant main effects of ischemia (F-statistic = 304.8888, p < 0.001) across all coronary arteries, indicating that ischemia significantly influences CAC scores. The main effect of collateral circulation was not significant (F-statistic = 1.0058e-11, p = 1.0), suggesting that collateral circulation does not independently affect CAC scores. However, the interaction effect between ischemia and collateral circulation was highly significant (F-statistic = 36.4755, p < 0.001), highlighting that the relationship between ischemia and CAC scores is strongly influenced by the presence of collateral circulation. These findings emphasize the importance of considering both ischemia and collateral circulation together in clinical assessments of CAD (Figure 10).

**Figure 11:**
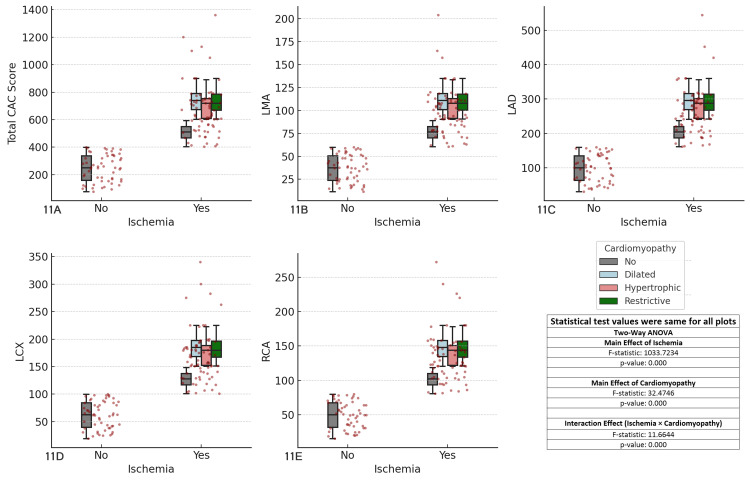
Comparison of coronary artery calcium (CAC) scores across major coronary arteries, ischemia status, and cardiomyopathy types Each boxplot compares the distribution of CAC scores for the specified coronary artery (total CAC, LMA, LAD, LCX, RCA) between patients with and without ischemia (Yes/No), considering cardiomyopathy as a factor. The boxplots display the interquartile range (IQR), with the median indicated, and individual data points overlaid as red dots. The two-way ANOVA test was used for comparisons. (A) Total CAC score vs ischemia and cardiomyopathy, (B) left main artery (LMA) CAC score vs ischemia and cardiomyopathy, (C) left anterior descending artery (LAD) CAC score vs ischemia and cardiomyopathy, (D) left circumflex artery (LCX) CAC score vs ischemia and cardiomyopathy, (E) right coronary artery (RCA) CAC score vs ischemia and cardiomyopathy.

The results revealed significant main effects of ischemia (F-statistic = 1033.7234, p < 0.001) across all coronary arteries. The main effect of cardiomyopathy was also significant (F-statistic = 32.4476, p < 0.001), indicating that different cardiomyopathy subtypes influence CAC scores. Furthermore, the interaction effect between ischemia and cardiomyopathy was significant (F-statistic = 11.6644, p < 0.001), suggesting that ischemia has varying impacts on CAC scores depending on the type of cardiomyopathy. Patients with dilated cardiomyopathy tended to exhibit the highest CAC scores, particularly in ischemic regions, followed by those with hypertrophic and restrictive cardiomyopathies, who showed comparatively lower scores. These findings suggest that the type of cardiomyopathy may play a crucial role in coronary artery calcification, emphasizing the need for tailored risk stratification and management, particularly for ischemic patients with dilated cardiomyopathy (Figure 11).

**Figure 12:**
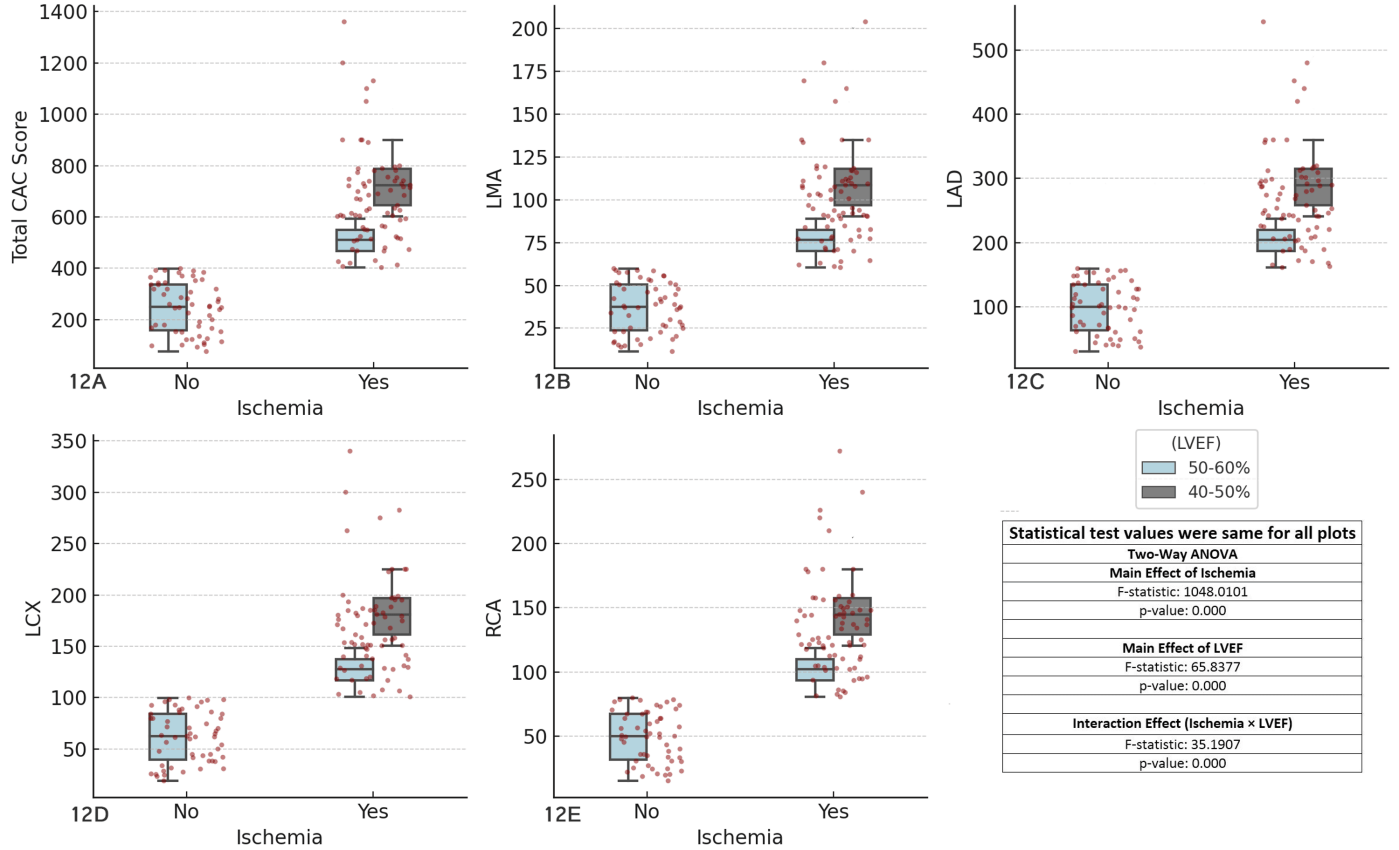
Comparison of coronary artery calcium (CAC) scores across major coronary arteries, ischemia status, and left ventricular ejection fraction (LVEF) Each boxplot compares the distribution of CAC scores for the specified coronary artery (total CAC, LMA, LAD, LCX, RCA) between patients with and without ischemia (Yes/No), considering left ventricular ejection fraction (LVEF) as a factor. The boxplots display the interquartile range (IQR), with the median indicated, and individual data points overlaid as red dots. The two-way ANOVA test was used for comparisons. (A) Total CAC score vs ischemia and LVEF, (B) left main artery (LMA) CAC score vs ischemia and LVEF, (C) left anterior descending artery (LAD) CAC score vs ischemia and LVEF, (D) left circumflex artery (LCX) CAC score vs ischemia and LVEF, (E) right coronary artery (RCA) CAC score vs ischemia and LVEF.

The results revealed significant main effects of ischemia (F-statistic = 1048.0101, p < 0.001) and LVEF (F-statistic = 65.8377, p < 0.001) across all coronary arteries, indicating that both ischemia and LVEF independently influence CAC scores. Additionally, the interaction effect between ischemia and LVEF was highly significant (F-statistic = 35.1907, p < 0.001), suggesting that the impact of ischemia on CAC scores varies depending on LVEF levels. Patients with lower LVEF (40-50%) tended to exhibit higher CAC scores, particularly in ischemic areas, compared with those with higher LVEF (50-60%). These findings emphasize the importance of considering both ischemia and LVEF in the clinical assessment of CAD, as the interaction between these factors significantly influences CAC scores (Figure 12).

**Figure 13:**
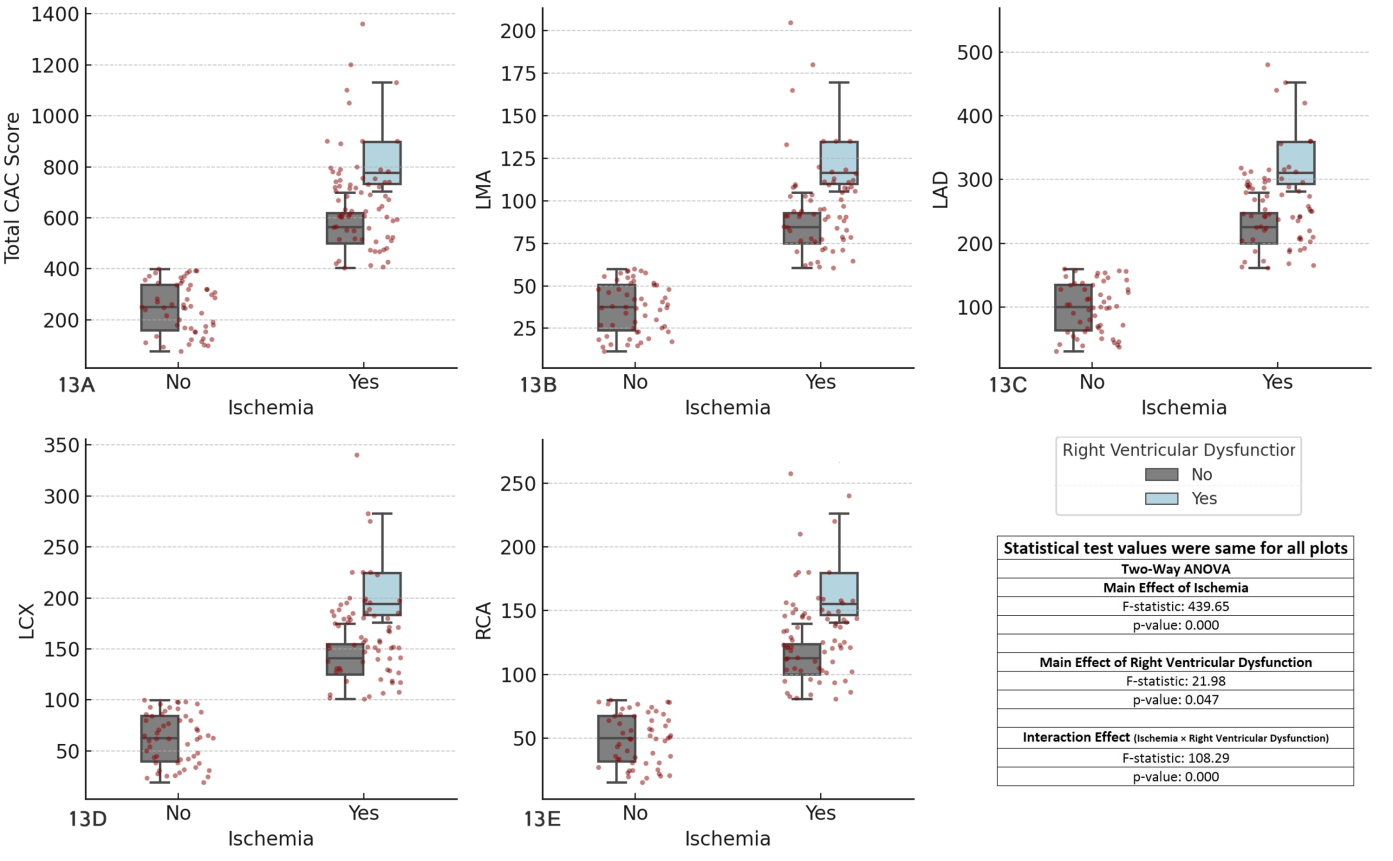
Comparison of coronary artery calcium (CAC) scores across major coronary arteries, ischemia status, and right ventricular dysfunction Each boxplot compares the distribution of CAC scores for the specified coronary artery (total CAC, LMA, LAD, LCX, RCA) between patients with and without ischemia (Yes/No), considering right ventricular dysfunction as a factor. The boxplots display the interquartile range (IQR), with the median indicated, and individual data points overlaid as red dots. The two-way ANOVA test was used for comparisons. (A) Total CAC score vs ischemia and right ventricular dysfunction, (B) left main artery (LMA) CAC score vs ischemia and right ventricular dysfunction, (C) left anterior descending artery (LAD) CAC score vs ischemia and right ventricular dysfunction, (D) left circumflex artery (LCX) CAC score vs ischemia and right ventricular dysfunction, (E) right coronary artery (RCA) CAC score vs ischemia and right ventricular dysfunction.

The results revealed significant main effects of ischemia (F-statistic = 439.6594, p < 0.001) across all coronary arteries, indicating that ischemia strongly influences CAC scores. The main effect of right ventricular dysfunction was also significant (F-statistic = 21.98, p = 0.047), suggesting that right ventricular dysfunction affects CAC scores. Additionally, the interaction effect between ischemia and right ventricular dysfunction was highly significant (F-statistic = 108.2949, p < 0.001), indicating that the relationship between ischemia and CAC scores is significantly modified by the presence of right ventricular dysfunction. These findings highlight the importance of considering both ischemia and right ventricular dysfunction in the clinical assessment of CAD, as their interaction plays a crucial role in determining CAC scores (Figure 13).

**Figure 14:**
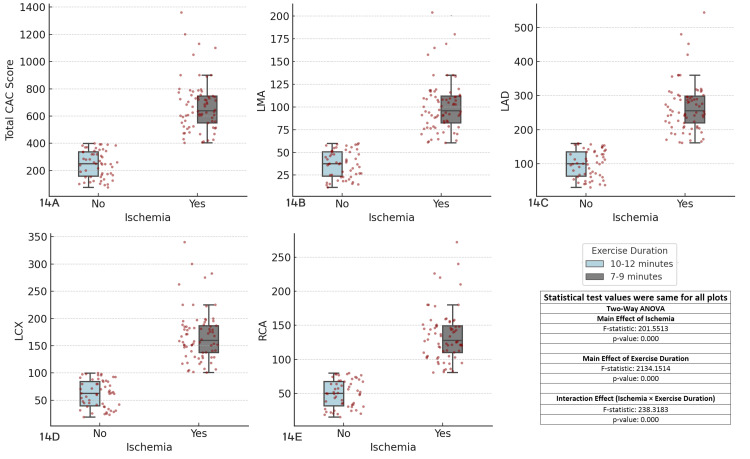
Comparison of coronary artery calcium (CAC) scores across major coronary arteries, ischemia status, and exercise duration Each boxplot compares the distribution of CAC scores for the specified coronary artery (total CAC, LMA, LAD, LCX, RCA) between patients with and without ischemia (Yes/No), considering exercise duration as a factor. The boxplots display the interquartile range (IQR), with the median indicated, and individual data points overlaid as red dots. The two-way ANOVA test was used for comparisons. (A) Total CAC score vs ischemia and exercise duration, (B) left main artery (LMA) CAC score vs ischemia and exercise duration, (C) left anterior descending artery (LAD) CAC score vs ischemia and exercise duration, (D) left circumflex artery (LCX) CAC score vs ischemia and exercise duration, (E) right coronary artery (RCA) CAC score vs ischemia and exercise duration.

The results revealed significant main effects of ischemia (F-statistic = 201.5513, p < 0.001) and exercise duration (F-statistic = 2134.1514, p < 0.001) across all coronary arteries, indicating that both ischemia and exercise duration independently influence CAC scores. Furthermore, the interaction effect between ischemia and exercise duration was highly significant (F-statistic = 238.3183, p < 0.001), suggesting that the impact of ischemia on CAC scores varies based on exercise duration. Patients with longer exercise durations (10-12 minutes) tended to exhibit higher CAC scores, particularly in ischemic regions, compared with those with shorter exercise durations (7-9 minutes). These findings emphasize the need to consider both ischemia and exercise duration in clinical assessments of CAD, as their interaction plays a crucial role in determining CAC scores (Figure 14).

**Figure 15:**
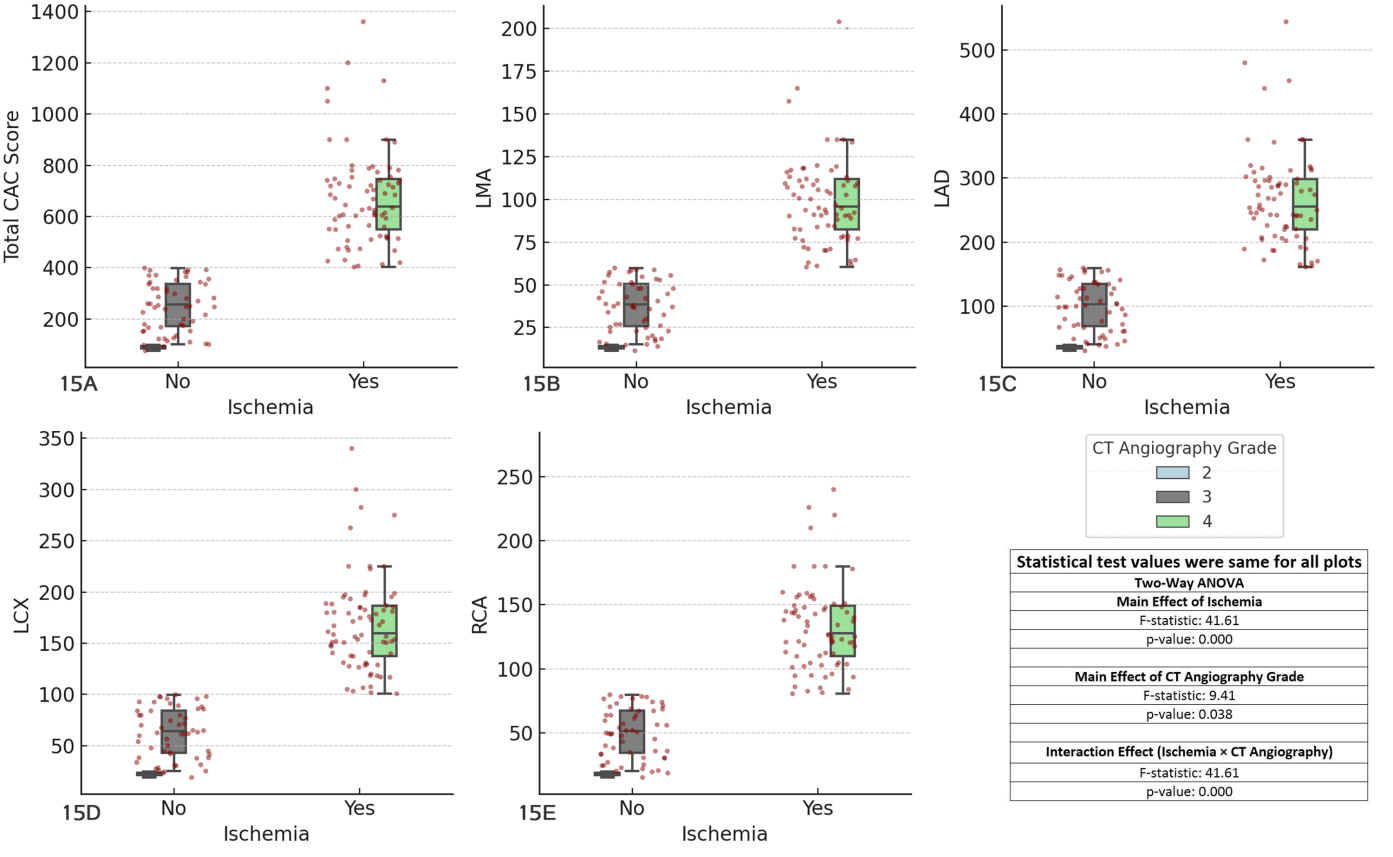
Comparison of coronary artery calcium (CAC) scores across major coronary arteries, ischemia status, and CT angiography grades Each boxplot compares the distribution of CAC scores for the specified coronary artery (total CAC, LMA, LAD, LCX, RCA) between patients with and without ischemia (Yes/No), considering CT angiography grades as a factor. The boxplots display the interquartile range (IQR), with the median indicated, and individual data points overlaid as red dots. The two-way ANOVA test was used for comparisons. (A) Total CAC score vs ischemia and CT angiography grades, (B) left main artery (LMA) CAC score vs ischemia and CT angiography grades, (C) left anterior descending artery (LAD) CAC score vs ischemia and CT angiography grades, (D) left circumflex artery (LCX) CAC score vs ischemia and CT angiography grades, (E) right coronary artery (RCA) CAC score vs ischemia and CT angiography grades.

The results revealed significant main effects of ischemia (F-statistic = 41.6141, p < 0.001) across all coronary arteries, indicating that ischemia significantly influences CAC scores. The main effect of CT angiography grade was also significant (F-statistic = 9.41, p = 0.038), suggesting that the severity of CAD, as graded by CT angiography, affects CAC scores. Additionally, the interaction effect between ischemia and CT angiography grade was highly significant (F-statistic = 41.6141, p < 0.001), indicating that the effect of ischemia on CAC scores is influenced by the severity of CAD. These findings suggest that both ischemia and CT angiography grade are important factors in determining CAC scores, highlighting the need to consider both when assessing CAD (Figure 15).

**Figure 16:**
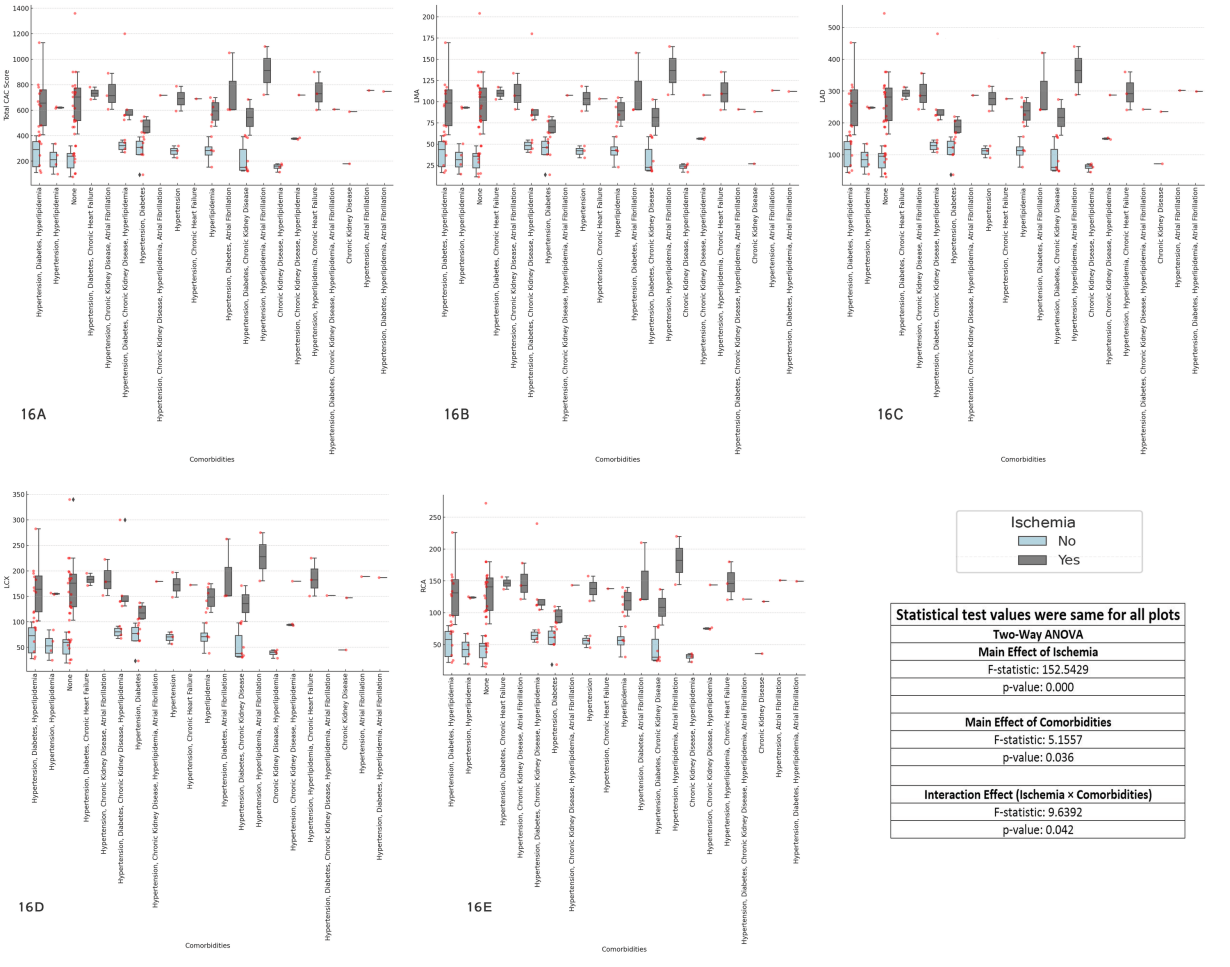
Impact of comorbidities on coronary artery calcium (CAC) scores and ischemia status across major coronary arteries Each boxplot compares the distribution of CAC scores for the specified coronary artery (total CAC, LMA, LAD, LCX, RCA) between patients with and without ischemia (Yes/No), considering comorbidities as a factor. The boxplots display the interquartile range (IQR), with the median indicated, and individual data points overlaid as red dots. The two-way ANOVA test was used for comparisons. (A) Comorbidities and ischemia vs total CAC score, (B) comorbidities and ischemia vs left main artery (LMA) CAC score, (C) comorbidities and ischemia vs left anterior descending artery (LAD) CAC score, (D) comorbidities and ischemia vs left circumflex artery (LCX) CAC score, (E) comorbidities and ischemia vs right coronary artery (RCA) CAC score.

The results revealed significant main effects of ischemia (F-statistic = 152.5429, p < 0.001) across all coronary arteries, indicating that ischemia strongly influences CAC scores. The main effect of comorbidities was also significant (F-statistic = 3.1557, p = 0.036), suggesting that comorbidities affect CAC scores. Additionally, the interaction effect between ischemia and comorbidities was significant (F-statistic = 9.6392, p = 0.042), indicating that the effect of ischemia on CAC scores is influenced by the presence of comorbidities. These findings emphasize the importance of considering both ischemia and comorbidities in the clinical assessment of CAD, as their interaction plays a crucial role in determining CAC scores (Figure 16).

**Figure 17:**
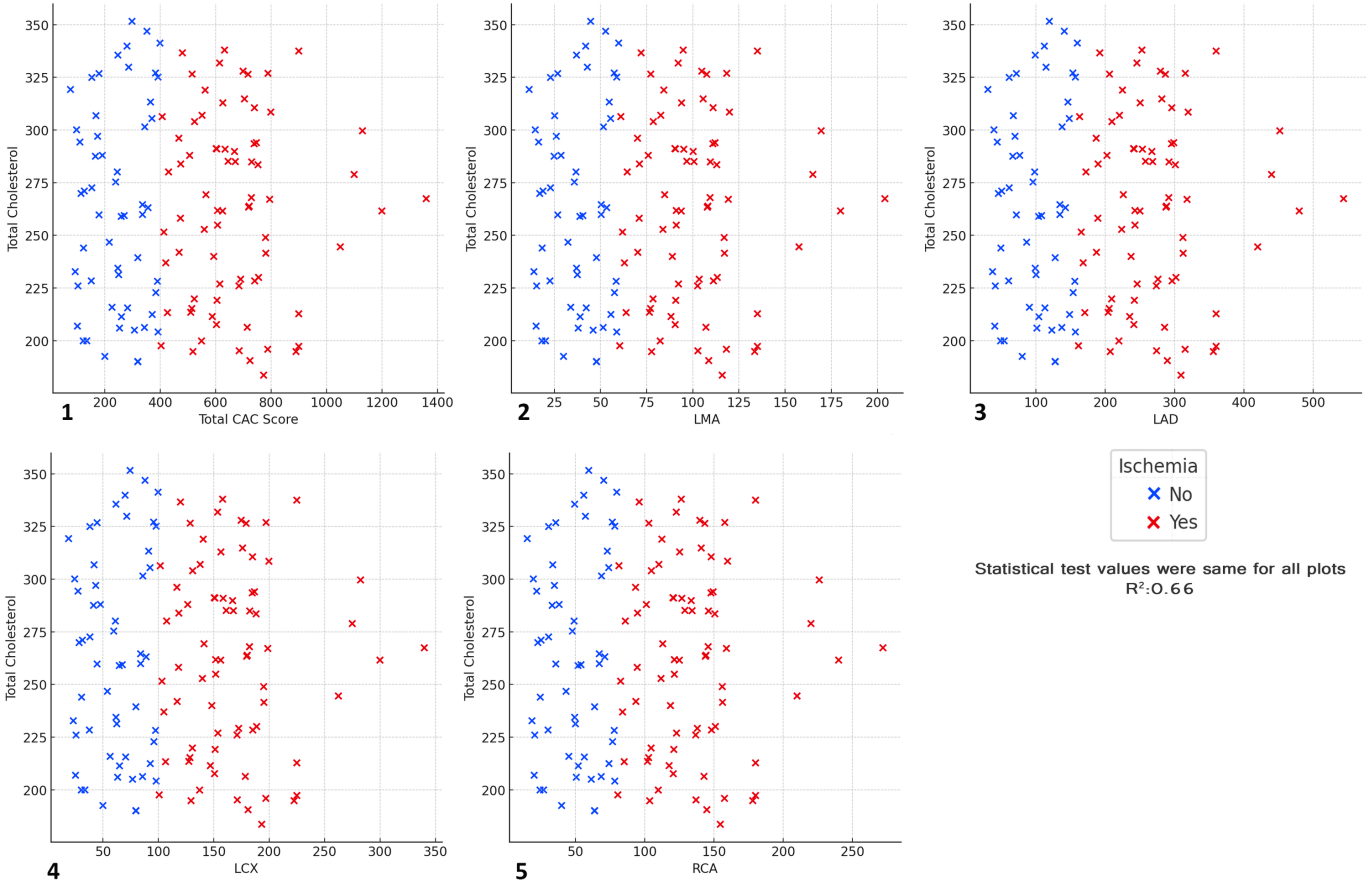
Relationship between total CAC score, major coronary arteries, total cholesterol, and ischemia status Scatter plots depict the relationship between total CAC score, major coronary arteries, total cholesterol, and ischemia status (R² = 0.66). (1) Total CAC score vs total cholesterol vs ischemia, (2) left main artery (LMA) vs total cholesterol vs ischemia, (3) left anterior descending artery (LAD) vs total cholesterol vs ischemia, (4) left circumflex artery (LCX) vs total cholesterol vs ischemia, (5) right coronary artery (RCA) vs total cholesterol vs ischemia.

The results indicated a moderate positive correlation (R² = 0.66) between total cholesterol levels and CAC scores across all arteries, with ischemic patients (represented by red) generally exhibiting higher CAC scores compared with non-ischemic patients (represented by blue). In each plot, as total cholesterol levels increased, there was a noticeable trend of increasing CAC scores, particularly in the ischemic group, suggesting that elevated cholesterol levels are associated with greater coronary calcification. This relationship was consistent across all coronary arteries, with the highest correlation observed in the total CAC score, followed by the LMA, LAD, LCX, and RCA. These findings emphasize the importance of monitoring cholesterol levels as part of cardiovascular risk assessment, as higher cholesterol levels are linked to increased coronary artery calcification and a greater risk of ischemic events. The results further underscore the need for cholesterol management to prevent the progression of CAD and ischemia (Figure 17).

**Figure 18:**
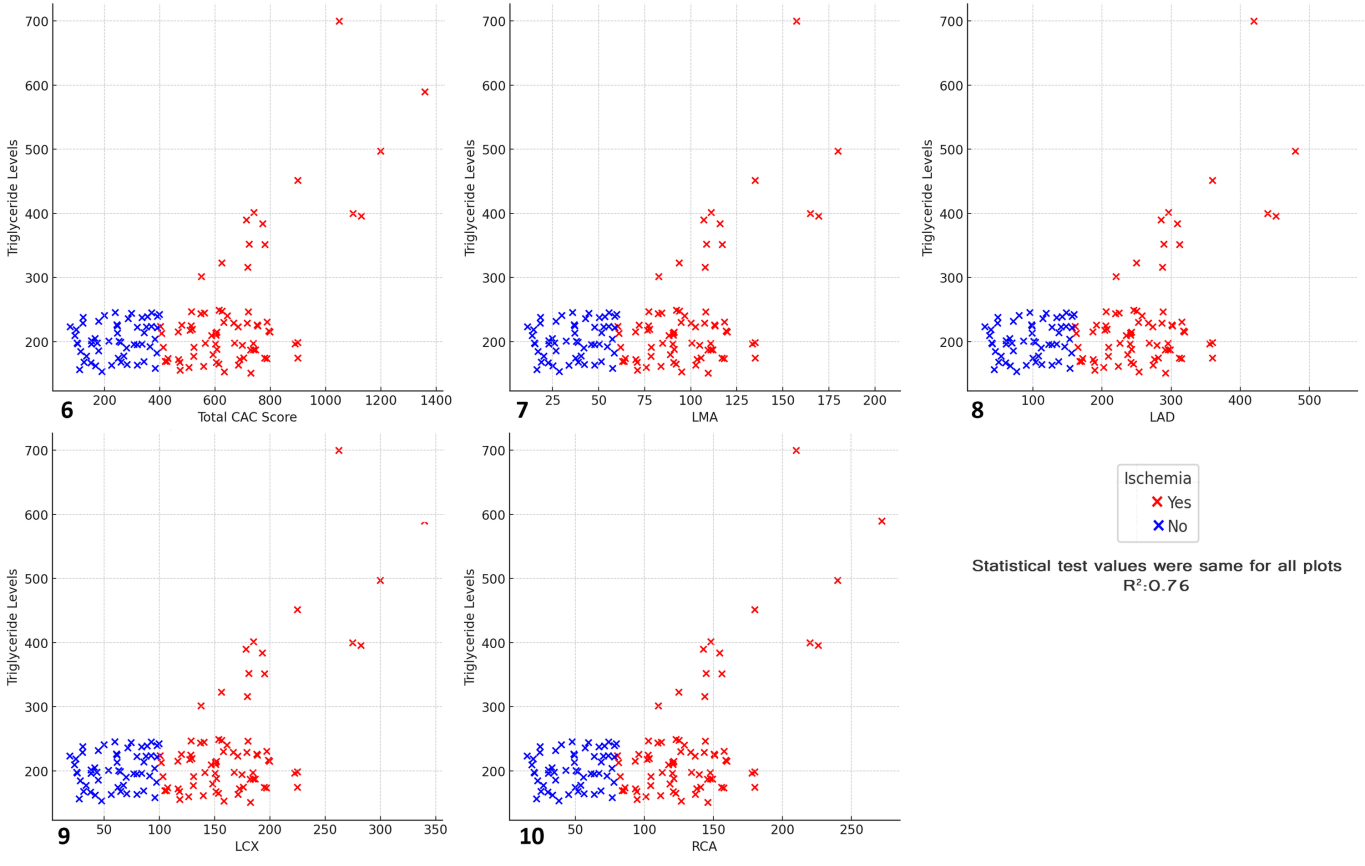
Relationship between total CAC score, major coronary arteries, triglyceride levels, and ischemia status Scatter plots depict the relationship between total CAC score, major coronary arteries, triglyceride levels, and ischemia status (R² = 0.76). (6) Total CAC score vs triglyceride levels vs ischemia, (7) left main artery (LMA) vs triglyceride levels vs ischemia, (8) left anterior descending artery (LAD) vs triglyceride levels vs ischemia, (9) left circumflex artery (LCX) vs triglyceride levels vs ischemia, (10) right coronary artery (RCA) vs triglyceride levels vs ischemia.

The results revealed a moderate to strong positive correlation (R² = 0.76) between triglyceride levels and CAC scores across all arteries. Ischemic patients (marked in red) generally exhibited higher triglyceride levels and CAC scores compared with non-ischemic patients (marked in blue). As triglyceride levels increased, there was a noticeable trend of higher CAC scores, particularly in ischemic individuals, suggesting that elevated triglyceride levels are associated with increased coronary calcification. This correlation was consistent across the total CAC score, LMA, LAD, LCX, and RCA, highlighting triglycerides as an important lipid marker for cardiovascular health. These findings suggest that high triglyceride levels contribute to the development of CAD and ischemia. Managing triglyceride levels could potentially help reduce coronary calcification and the associated risk of ischemic events (Figure 18).

**Figure 19:**
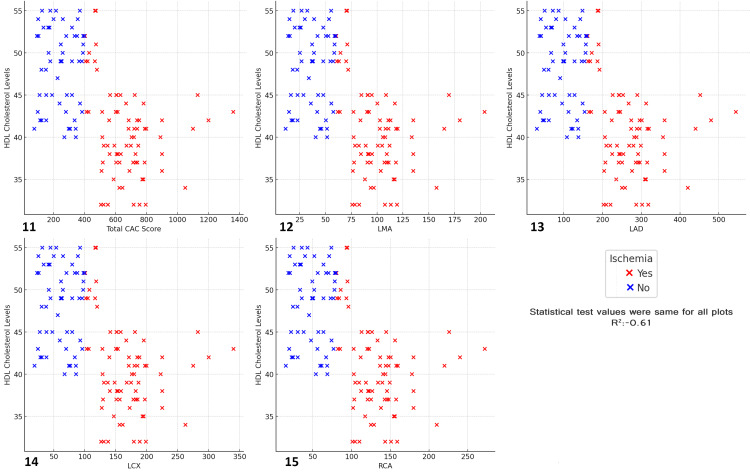
Relationship between total CAC score, major coronary arteries, HDL cholesterol levels, and ischemia status Scatter plots depict the relationship between total CAC score, major coronary arteries, HDL cholesterol levels, and ischemia status (R² = -0.61). (11) Total CAC score vs HDL cholesterol levels vs ischemia, (12) left main artery (LMA) vs HDL cholesterol levels vs ischemia, (13) left anterior descending artery (LAD) vs HDL cholesterol levels vs ischemia, (14) left circumflex artery (LCX) vs HDL cholesterol levels vs ischemia, (15) right coronary artery (RCA) vs HDL cholesterol levels vs ischemia.

The results revealed a moderate negative correlation (R² = -0.61) between HDL cholesterol levels and CAC scores. Ischemic patients (represented by red) tended to have lower HDL cholesterol levels and higher CAC scores compared with non-ischemic patients (represented by blue). As HDL cholesterol levels decreased, there was a noticeable increase in CAC scores, especially in ischemic patients, suggesting that lower HDL cholesterol levels are associated with greater coronary calcification. This trend was consistent across all coronary arteries, including the total CAC score, LMA, LAD, LCX, and RCA. These findings highlight the protective role of HDL cholesterol in cardiovascular health, as higher HDL levels may help mitigate coronary artery calcification and reduce the risk of ischemic events. Managing HDL cholesterol levels could be a crucial part of strategies to prevent CAD and ischemia (Figure 19).

**Figure 20:**
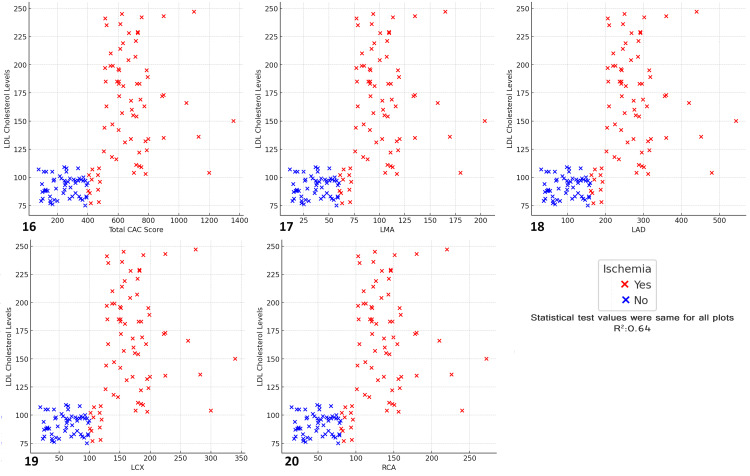
Relationship between total CAC score, major coronary arteries, LDL cholesterol levels, and ischemia status Scatter plots depict the relationship between total CAC score, major coronary arteries, LDL cholesterol levels, and ischemia status (R² = 0.64). (16) Total CAC score vs LDL cholesterol levels vs ischemia, (17) left main artery (LMA) vs LDL cholesterol levels vs ischemia, (18) left anterior descending artery (LAD) vs LDL cholesterol levels vs ischemia, (19) left circumflex artery (LCX) vs LDL cholesterol levels vs ischemia, (20) right coronary artery (RCA) vs LDL cholesterol levels vs ischemia.

The results revealed a moderate positive correlation (R² = 0.64) between LDL cholesterol levels and CAC scores across all arteries. Ischemic patients (represented by red) generally exhibited higher LDL cholesterol levels and higher CAC scores compared with non-ischemic patients (represented by blue). As LDL cholesterol levels increased, there was a corresponding rise in CAC scores, especially in ischemic individuals, indicating that elevated LDL cholesterol is associated with increased coronary calcification. This trend was evident across all coronary arteries, including the total CAC score, LMA, LAD, LCX, and RCA. These findings suggest that high LDL cholesterol levels are a significant contributor to coronary artery calcification, which in turn increases the risk of ischemic events. Managing LDL cholesterol could be crucial in reducing the progression of CAD and preventing ischemia (Figure 20).

## Discussion

The primary objective of this study was to examine the relationship between CAC scores and the development of myocardial ischemia, focusing on how demographic, clinical, and cardiovascular risk factors influence this relationship. A cohort of 129 patients, with a mean age of 59.64 ± 9.43 years, was included, of which 74 (57.36%) showed signs of myocardial ischemia. The study identified significant associations between elevated CAC scores and ischemia, supporting previous research that emphasizes the predictive value of CAC scores for cardiovascular outcomes [24]. In addition, clinical factors such as age, body mass index (BMI), smoking history, and comorbidities were found to impact coronary artery calcification and the likelihood of ischemia [25,26].

The findings align with literature that supports the use of CAC scoring as a predictor of CAD and myocardial ischemia [27]. The CAC score was significantly higher in ischemic patients, demonstrating a strong link between coronary calcification and ischemia, which is consistent with studies associating higher CAC scores with an increased risk of MI and ischemic events [28,29].

This study also identified specific CAC score thresholds for predicting myocardial ischemia, such as a total CAC score of 403.0, as well as thresholds for individual arteries, including 161.2 for the LAD, 100.75 for the LCX, 80.6 for the RCA, and 60.45 for the LMA. These thresholds exhibited moderate to strong predictive power, as indicated by Gini indices ranging from 0.44 to 0.59. This finding supports the clinical relevance of CAC scoring in guiding risk stratification and decisions about preventive interventions [30,31]. These results are consistent with the American College of Cardiology/American Heart Association (ACC/AHA) guidelines, which provide a Class II recommendation for the use of CAC scoring as a reasonable adjunct to cardiovascular risk stratification, particularly in cases where traditional risk factors do not fully capture an individual’s risk profile [32]. This further reinforces the clinical utility of CAC scoring as highlighted in this study.

The impact of age on coronary artery calcification is well established. Our study found that patients in the 60-70 and ≥70 years age groups had higher CAC scores, particularly among those with ischemia. This aligns with prior research showing that older individuals tend to have more extensive coronary calcification and a heightened risk of ischemic events [33,34]. The higher CAC scores in older patients suggest that age is a critical factor in the progression of CAD, supporting the need for age-based risk stratification when evaluating ischemia.

Previous research has explored gender differences in CAC scores, typically finding that men show higher CAC scores [35]. However, our study found that gender did not significantly influence CAC scores across the different coronary arteries, as the main effect of gender was not significant (F-statistic = 0.1646, p = 0.6857). While males had higher CAC scores overall, particularly among ischemic patients, this did not show a notable difference when compared with females. These findings suggest that while ischemia plays a crucial role in CAD progression, gender does not appear to require distinct risk stratification based on CAC scores in the studied coronary arteries. Thus, the focus on ischemia remains paramount in risk assessment and prevention strategies.

The link between obesity and CAD is further supported by this study, which found that higher BMI is associated with increased coronary calcification [36]. In our study, overweight and obese patients had the highest CAC scores, particularly among those with ischemia. This finding highlights the importance of weight management as part of cardiovascular risk reduction, as reducing BMI could potentially lower coronary calcification and the risk of ischemic events.

Smoking remains a significant modifiable risk factor for CAD [37], and this study confirms its detrimental effects on coronary artery calcification. Smokers had significantly higher CAC scores compared with non-smokers, particularly among ischemic patients. This emphasizes the need for smoking cessation as a critical component of cardiovascular disease prevention, as reducing smoking rates could lower coronary calcification and improve overall cardiovascular health.

The role of comorbid conditions such as hypertension, diabetes, and hyperlipidemia in exacerbating CAD [38] is highlighted in this study. Patients with ischemia and comorbidities had higher CAC scores across all coronary arteries. This finding supports previous research indicating that comorbidities accelerate atherosclerosis and increase ischemia risk [39,40]. Managing these conditions is crucial for reducing coronary calcification and preventing ischemic events. Comprehensive strategies that address both traditional risk factors and comorbidities are essential to optimizing patient outcomes.

The association between CAC scores and MI was confirmed in this study. Patients with ischemia and a history of MI had higher CAC scores compared with those without a prior MI [41]. This emphasizes the role of CAC scoring in identifying patients at high risk for future MI and ischemic events, underscoring its potential in clinical decision-making and risk stratification.

While this study provides valuable insights into the relationship between CAC scores and myocardial ischemia, several important limitations should be acknowledged. First, the cross-sectional design precludes conclusions about causality, and the findings should be interpreted as associative rather than causal. Longitudinal studies are needed to clarify the temporal relationship between coronary calcification and ischemic outcomes. Second, the use of convenience sampling may introduce selection bias, limiting the generalizability of the results to broader populations. Third, there was heterogeneity in ischemia assessment, as multiple diagnostic modalities were employed without uniform adjudication, which could influence consistency in outcome classification. Fourth, precise interval data between CAC scoring and ischemia testing were not systematically collected due to the difficulty of reliably estimating this from patient claims and clinical records; nevertheless, all patients underwent both evaluations within the same clinical assessment period. Fifth, the study did not include long-term follow-up or outcome data, which restricts the ability to determine the prognostic significance of the CAC thresholds identified. Finally, while significant associations were observed between CAC scores and several risk factors, the underlying pathophysiological mechanisms remain unclear. Accordingly, the present findings should be viewed as hypothesis-generating, warranting validation in larger, prospective, and multicenter studies to refine risk stratification models and assess clinical utility.

## Conclusions

This study underscores the significant role of CAC scoring in assessing the risk of myocardial ischemia and cardiovascular events. Elevated CAC scores are strongly linked with ischemia, particularly in individuals with risk factors such as age, BMI, smoking, and comorbidities. By integrating CAC scoring into clinical practice, clinicians can more effectively identify high-risk patients and guide decision-making for targeted interventions. The identification of specific CAC score thresholds provides valuable benchmarks for improved stratification of patients based on their risk for ischemia. Combining CAC scores with traditional risk factors enhances early detection of individuals at higher risk, enabling more personalized and focused treatment approaches. Ultimately, this approach has the potential to improve patient outcomes by facilitating precise risk assessment and optimizing treatment strategies in patient management.

## Appendices

Data collection using Google Forms

1. Participant Information

Full Name (Optional): (Text entry)

Age: (Text entry)

Gender: (Multiple choice)

 Male

 Female

2. Body Mass Index (BMI)

(Text entry)

3. Smoking History

Do you smoke? (Multiple choice)

 Yes

 No

4. Comorbidities

(Text entry)

5. Cardiac Conditions

Ischemia: (Multiple choice)

 Yes

 No

History of Myocardial Infarction: (Multiple choice)

 Yes

 No

Collateral Circulation: (Multiple choice)

 Yes

 No

Cardiomyopathy Type: (Multiple choice)

 No Cardiomyopathy

 Restrictive

 Dilated

 Hypertrophic

Valvular Heart Disease: (Multiple choice)

 Yes

 No

6. Left Ventricular Ejection Fraction (LVEF)

LVEF Measurement: (Multiple choice)

 50-60%

 40-50%

7. Right Ventricular Dysfunction

Do you have evidence of right ventricular dysfunction? (Multiple choice)

 Yes

 No

8. Exercise Duration

How long can you exercise or undergo pharmacological stress? (Multiple choice)

 7-9 minutes

 10-12 minutes

9. CT Angiograph Grades

CT Angiography Grade: (Multiple choice)

 Grade 1

 Grade 2

 Grade 3

 Grade 4

10. Coronary Artery Calcium (CAC) Scores

Total Coronary Artery Calcium Score: (Text entry)

Left Main Artery (LMA) CAC Score: (Text entry)

Left Anterior Descending Artery (LAD) CAC Score: (Text entry)

Left Circumflex Artery (LCX) CAC Score: (Text entry)

Right Coronary Artery (RCA) CAC Score: (Text entry)

11. Lipid Profile

Total Cholesterol (mg/dL): (Text entry)

Triglyceride Levels (mg/dL): (Text entry)

HDL Cholesterol Levels (mg/dL): (Text entry)

LDL Cholesterol Levels (mg/dL): (Text entry)

12. Consent Confirmation

I give my informed consent to participate in this study: (Checkbox)

Yes
